# Investigating the mechanism of hepatocellular carcinoma progression by constructing genetic and epigenetic networks using NGS data identification and big database mining method

**DOI:** 10.18632/oncotarget.13100

**Published:** 2016-11-04

**Authors:** Cheng-Wei Li, Ping-Yao Chang, Bor-Sen Chen

**Affiliations:** ^1^ Laboratory of Control and Systems Biology, National Tsing Hua University, Hsinchu, Taiwan

**Keywords:** DNA methylation, multiple potential drugs, hepatocarcinogenesis, miRNAs, principal network projection

## Abstract

The mechanisms leading to the development and progression of hepatocellular carcinoma (HCC) are complicated and regulated genetically and epigenetically. The recent advancement in high-throughput sequencing has facilitated investigations into the role of genetic and epigenetic regulations in hepatocarcinogenesis. Therefore, we used systems biology and big database mining to construct genetic and epigenetic networks (GENs) using the information about mRNA, miRNA, and methylation profiles of HCC patients. Our approach involves analyzing gene regulatory networks (GRNs), protein-protein networks (PPINs), and epigenetic networks at different stages of hepatocarcinogenesis. The core GENs, influencing each stage of HCC, were extracted via principal network projection (PNP). The pathways during different stages of HCC were compared. We observed that extracellular signals were further transduced to transcription factors (TFs), resulting in the aberrant regulation of their target genes, in turn inducing mechanisms that are responsible for HCC progression, including cell proliferation, anti-apoptosis, aberrant cell cycle, cell survival, and metastasis. We also selected potential multiple drugs specific to prominent epigenetic network markers of each stage of HCC: lestaurtinib, dinaciclib, and perifosine against the *NTRK2*, *MYC*, and *AKT1* markers influencing HCC progression from stage I to stage II; celecoxib, axitinib, and vinblastine against the *DDIT3*, *PDGFB*, and *JUN* markers influencing HCC progression from stage II to stage III; and atiprimod, celastrol, and bortezomib against *STAT3*, *IL1B*, and *NFKB1* markers influencing HCC progression from stage III to stage IV.

## INTRODUCTION

Hepatocellular carcinoma (HCC) is the fifth most common type of cancer and the third leading cause of cancer-related deaths worldwide [1]. Understanding the molecular mechanism involved in the development and progression of HCC is imperative for the development of more efficacious therapeutic strategies, given that the worldwide incidence of HCC, a type of cancer with a poor 5-year survival rate (at the late stage) and high rate of recurrence (after surgical resection) [2], stands at over one million. The development and progression of HCC is a long-term multistep process, comprising chronic liver injury, necro-inflammation and regeneration, small cell dysplasia, and the appearance of low- and high-grade dysplastic nodules [3, 4].

Early research studies concluded that HCC is a result of the stepwise accumulation of genetic alterations in oncogenes and tumor suppressor genes, which in turn led to the regulation of cell proliferation, growth, survival, apoptosis, adhesion, and metabolism [5]. However, growing evidence has indicated the added involvement of DNA methylation and regulation of miRNA in the development of HCC [6]. Presently, HCC is recognized as a genetic and epigenetic disease; that is, both genetic and epigenetic components are believed to be involved in all stages of liver carcinogenesis [7].

DNA promoter methylation, an important process for transcriptional regulation and cellular differentiation, is a common mechanism of gene silencing in cancer cells [8]. Recent studies have specifically focused on cancer-linked gene-specific DNA hypermethylation or hypomethylation. According to the report in [7], several studies have indicated that some tumor suppressors, involved in the regulation of vital biological processes such as cell-cycle, apoptosis, and cell proliferation, are frequently methylated in HCC. On the other hand, tumor-promoting genes that play a critical role in cell growth, communication, adhesion and mobility, signal transduction, and drug resistance have also been shown to be significantly hypomethylated.

miRNAs are small, evolutionarily conserved, single-stranded molecules that are approximately 22 nucleotides in length, which regulate gene expression by binding to complementary sequences in the 3′ untranslated region (UTR) of target messenger RNA (mRNA); this generally results in their degradation or translational inhibition [9, 10]. More than 1000 miRNAs have been discovered in humans, each of which could target, on average, more than 200 genes [11, 12]. Recent reports suggest that miRNA play critical roles in HCC progression: for example, deregulation of miRNA could lead to irregular cell proliferation, evasion of apoptotic cell death, angiogenesis, and invasion and metastasis, by targeting a large number of protein-coding genes [13, 14]. In addition, detection of the level of miRNA in the blood plasma and serum could allow for earlier cancer diagnosis, accurate prediction of prognosis, and better response to therapy [15]. Despite recognizing their importance in regulating the expression of protein-coding genes, the precise functions of miRNA remain elusive [12].

A number of studies conducted over the past few years have implicated both genetic and epigenetic events in the development and progression of HCC; however, the results of most of these studies do not reach a consensus, given that cancer is a complex system. In fact, a recent review has suggested that many of these studies focus narrowly on the specific effect of a given miRNA on a specific mRNA, rather than the panoramic view of an extensive network [10].

In order to construct GRN or PPIN, correlation coefficients of expressions between genes remain the most used method to investigate hepatocarcinogenesis [16–18]. However, the correlation networks have undirected edges and no causality among whole genes. In this study, we applied a system identification method and a system order detection scheme to network models, which could characterize molecular mechanisms in gene regulatory network (GRN) and protein-protein interaction network (PPIN), to identify the real genetic and epigenetic network (GEN) using candidate GEN, NGS data, and DNA methylation profiles in normal liver cells and HCC.

Since the further analysis of a large-scale network is complex, the core network should be extracted from the real GEN to analyze the core mechanism of hepatocarcinogenesis. Up to now, the core network was still identified by the network hubs to investigate the core mechanism of carcinogenesis [18, 19]. Because we could identify the connection weights of the real GEN using genome-side expression data, we applied a principal network projection (PNP) method based on principal component analysis (PCA) to the real GEN to extract the core network from the viewpoint of significant large-scale network structure. By comparing two core networks from a perspective of signal transduction pathways, we suggested that the differences in epigenetic miRNA regulation and methylation may be the major factor involved in the mechanism of HCC progression. We have also suggested drug molecules specific for multiple target genes that are aberrantly methylated, or miRNA that are deregulated, in HCC, to prevent disease progression. Therefore, the proposed GEN construction and PNP method could be used to propose novel drug targets for HCC therapy, in addition to providing insight into the mechanism of HCC progression.

## RESULTS

**Figure 1:**
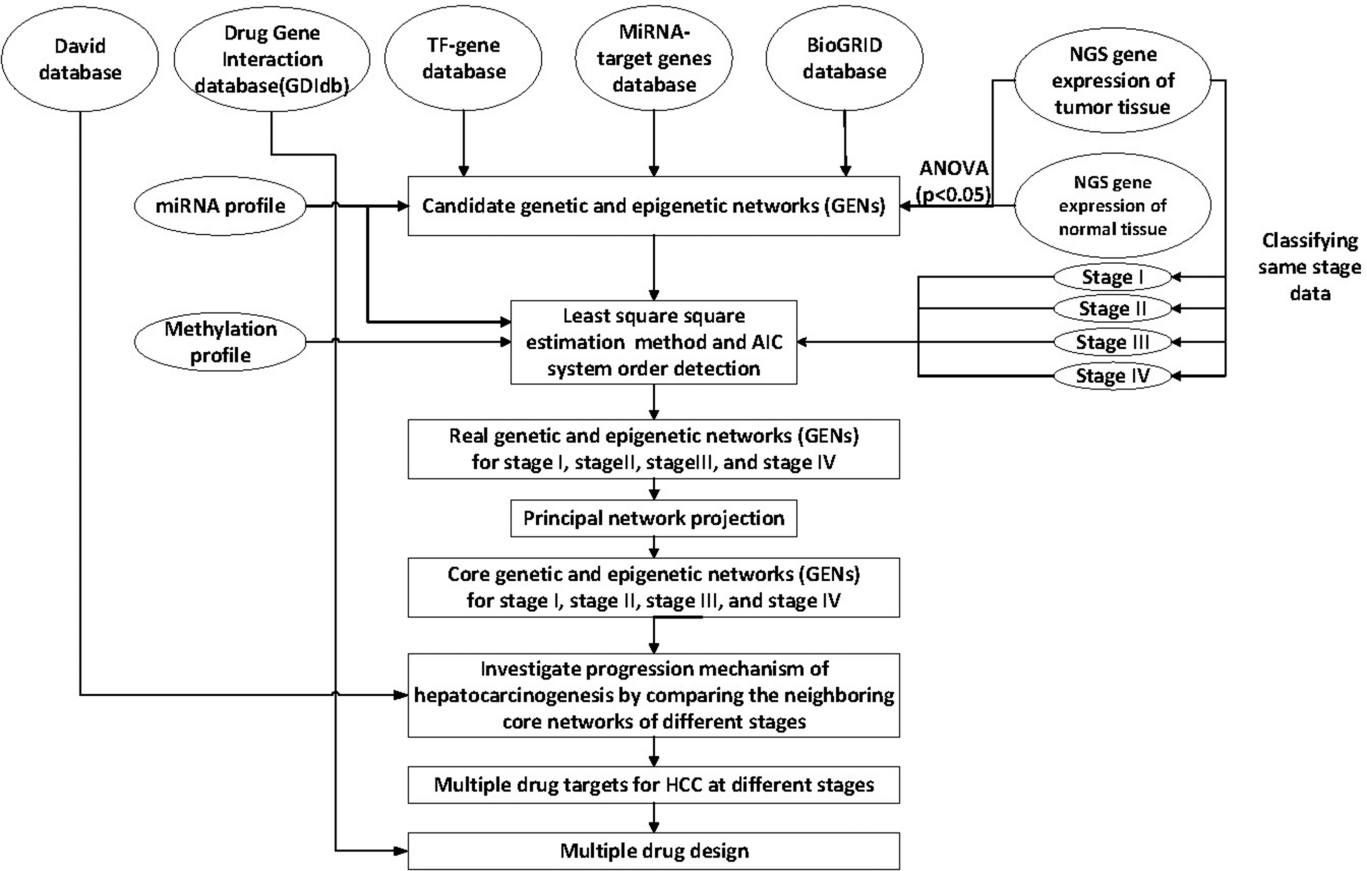
Overview of the construction of genetic and epigenetic networks (GENs) and core GEN to analyze the mechanism of hepatocarcinogenesis progression and multiple-drug design, via big database mining and system identification NGS profiles of HCC and normal tissue samples, miRNA and DNA methylation data, and the miRNA, TF-gene, and BioGRID databases were searched to construct candidate GENs, including candidate GRN, PPIN, and miRNA-gene regulatory networks. The false positive candidate GENs were pruned by system modeling and least square estimation, wherein the insignificant components were deleted from the system order based on AIC. The core GENs for each stage were obtained by extracting the principal structure of GENs through PNP. By mapping the core GENs to pathways retrieved using DAVID, and by comparing core GENs among the different stages, we investigated the mechanism of HCC progression. We also proposed potential therapeutic drugs against genes encoding significant epigenetic changes, in order to arrest the progression of HCC, by mining the DGIdb.

**Figure 2:**
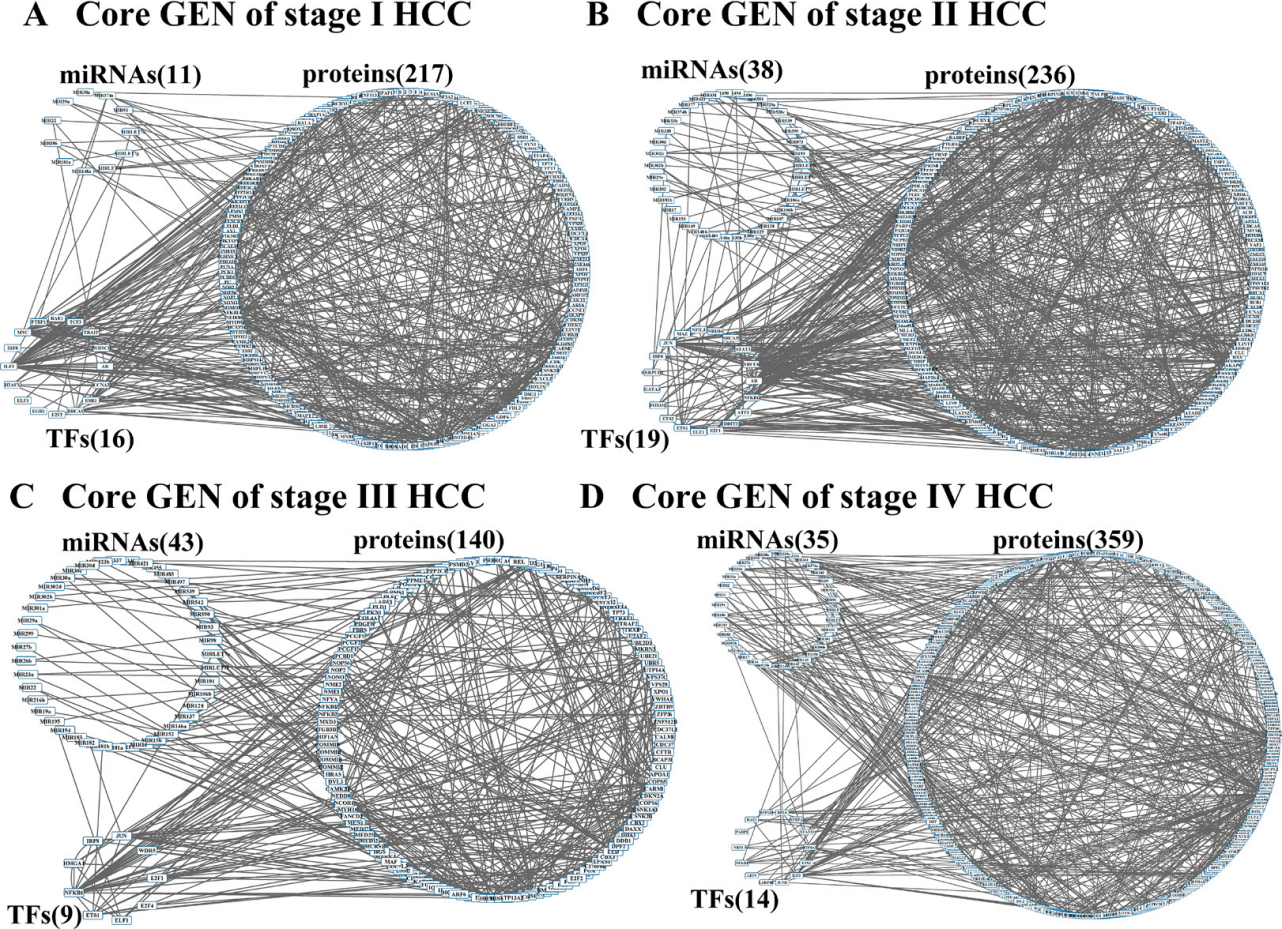
Core GENs of stage I (A), stage II (B), stage III (C), and stage IV (D) HCCs extracted by PNP method

The flowchart of the construction of GENs to analyze the mechanism of hepatocarcinogenesis progression and multiple-drug design is summarized in Figure 1. GENs of the various stages of HCC were constructed as shown in Figures S1–S4. PNP led to the extraction of four core GENs for each stage of HCC (Figure 2A–2D). In order to validate the results in this study, we used the validation set from a published microarray data set in [20] that included 102, 100, and 38 tissue samples from subjects with HCC stage I, II, and III, respectively. Because, in order to validate the real GRN by applying the model in (1), simultaneous measurement of gene expression and DNA methylation profiles in each tissue sample were required, we only validated the PPINs of the real GENs in HCC stage I, II, and III using the validation set. By comparing the real PPINs in Figures S1–S3 identified by using the data from TCGA with the real PPINs identified by using the validation set from [20], the results show 74.44%, 72.6%, and 71.08% polarity consistency of network edges, which were not pruned by AIC model order detection and have unchanged signs, in HCC stage I, II, and III, respectively. By also applying PNP to the real PPINs identified by the validation set in HCC stage I, II, and III, the projection distance in the PPINs identified by using validation set was highly correlated with the results in this study in HCC stage I (*R*^2^ = 0.909), II (*R*^2^ = 0.928), and III (*R*^2^ = 0.902) (Figure S5). Although the sample size of HCC stage III in the validation set, and HCC stage II and III in this study is smaller than 100, the proposed methods apply the system identification and system order detection to the system models of GEN in (1)–(2) to identify the real GEN and then apply PNP to the real GEN to provide robust results to extract the core GEN. These results support the notion that projection distance, as evaluation tools for the core GEN of the integrated genetic and epigenetic network, can achieve acceptable reliability in performance.

Furthermore, we plot *p*-value (log_10_) versus projection distance (log_2_) of each component (Figure S6) in HCC stage 1–4 (Figures S1–S4). Although the result in Figure S6 shows that the relationship between *p*-value (log_10_) and projection distance (log_2_) is not linear, the core GENs of HCC stages 1–4 (Figure 2A–2D) only have 13, 10, 5, and 41 components with insignificant *p*-value (> 0.05). It means that by applying the projection distance in (15) to the GEN of each HCC stage we can obtain the core GEN with a high proportion of differentially expressed genes (> 90%).

The pathways involved in the core PPIs of GENs were investigated using the DAVID (https://david.ncifcrf.gov/) and PANTHER tools, which were used to identify the pathways of the core proteins at each stage and to identify the biological processes of the target genes at each stage, respectively. From these results, pathways containing TFs in core proteins, which simultaneously have target genes in core genes, were selected to investigate the progression of HCC.

### Pathway analysis of core proteins and the impact of epigenetic miRNA and DNA methylation on core GEN of stage I HCC

The PNP method was used to isolate the core GEN of stage 1 HCC. The pathways regulated by the core proteins were identified and analyzed using the bioinformatics database DAVID, which analyzed the proteins and retrieved relevant results from the Kyoto Encyclopedia of Genes and Genomes (KEGG) database. By applying the criteria *p*-value ≤ 0.05 and the number of proteins ≥ 5, we can obtain the enriched pathways of core proteins in the GEN of each HCC stage. The results of this analysis are summarized in Table 1; the neurotrophin signaling, B cell receptor signaling, ErbB signaling, T cell receptor signaling, Wnt signaling, MAPK signaling, and chemokine signaling pathways and cancer-related pathways were found to be enriched.

**Table 1:** Pathway analysis of core proteins in the GEN of stage I HCC

Pathway analysis		
**KEGG pathway**	**Numbers**	***p*-value**
**Pathways in cancer**	16	1.4E-3
**MAPK signaling pathway**	12	9.0E-3
**Neurotrophin signaling pathway**	11	1.4E-4
**Wnt signaling pathway**	9	5.0E-3
**Chemokine signaling pathway**	9	3.0E-2
**ErbB signaling pathway**	8	3.5E-3
**B cell receptor signaling pathway**	8	6.1E-4
**T cell receptor signaling pathway**	8	1.7E-3

The enriched KEGG pathways in this GEN are the neurotrophin signaling pathway, B cell receptor signaling pathway, ErbB signaling pathway, pathways in cancer, T cell receptor signaling pathway, Wnt signaling pathway, MAPK signaling pathway, and chemokine signaling pathway.

**Figure 3:**
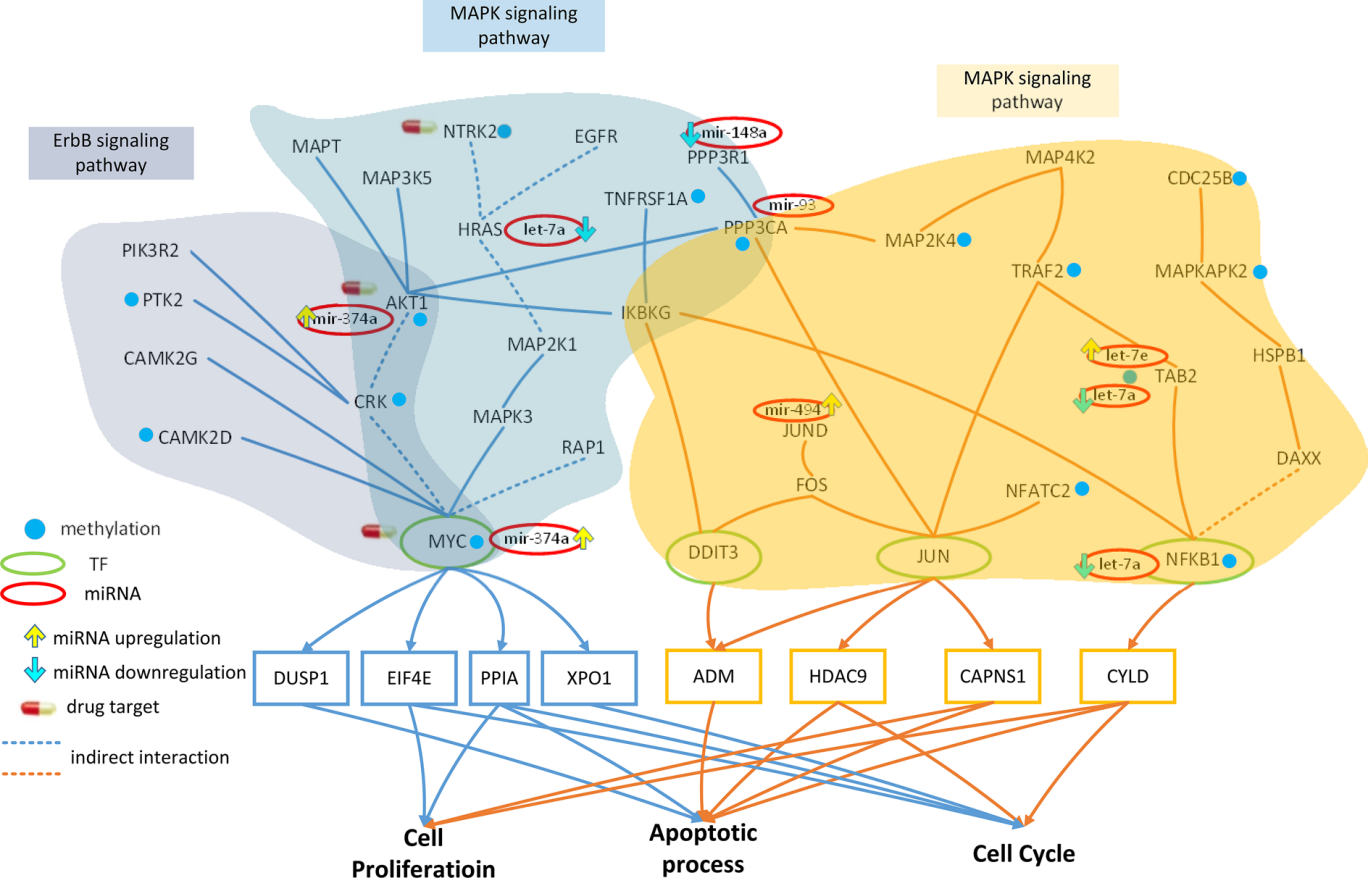
Effect of miRNA regulation and DNA methylation on the mechanism of progression of HCC from stage I to stage II The blue frames and lines represent stage I HCC, while the yellow frames and lines represent stage II HCC. Blue nodes represent DNA methylation. The green and red ovals represent transcription factors (TFs) and miRNAs, respectively. The upward yellow arrowhead represents miRNA upregulation during stage II HCC, compared to that in stage I. The downward blue arrowhead represents miRNA downregulation in stage II compared to that in stage I. *AKT1, RTK2, HRAS, CAMK2G, CAMK2D, PI3KR2, CRK,* and *MYC* are involved in the ErbB signaling pathway and *AKT1, TNFRSF1A, HRAS, MAP3K5, MAPT, IKBKG, NTRK2, PPP3R1, RAP1A, PPP3CA, CRK,* and *MYC* are involved in the MAPK signaling pathway during HCC stage I. The transcription factor *MYC* is activated by a number of stress signals, including the ErbB pathway and MAPK signaling pathway, which induce various cellular responses, including cell proliferation, apoptosis, and cell cycle. *TRAF2, MAP2K4, MAP4K2, NFKB1, MAPKAPK2, DAXX, TAB2, DIT3, CDC25B, FOS, JUN, JUND, IKBKG, HSPB1, PPP3CA*, and *NFATC2* regulate the MAPK signaling pathway during HCC stage II. The transcription factors *DDIT3*, *JUN*, and *NFKB1* are activated by a number of stress signals including the MAPK signaling pathway, which induces cell proliferation, apoptosis, and cell cycle. miRNA and DNA methylation induced dysregulation may contribute to the perturbation of the MAPK and ErbB pathway, resulting in aberrant cellular responses and HCC progression from stage I to stage II. Obviously, the shift from the ErbB pathway to the MAPK pathway, owing to the differences in epigenetic miRNA regulation and methylation could be responsible for disease progression. In this study, *NTRK2*, *CDC25B*, and *HRAS* were selected as potential drug targets to prevent the progression of HCC from stage I to stage II.

Some of these pathways, however, could influence downstream genes regulated by TFs. Therefore, we focused on pathways containing TFs in core proteins that have target genes in core genes. Figure 3 showed that both the ErbB and MAPK pathways contain MYC, a TF, which is encoded by target areas in core genes. This allowed us to analyze the effect of miRNA and DNA methylation on these pathways; we determined that dysfunctions in these processes might cause an aberration in the function of downstream target genes. This will be discussed in further detail in the Discussion section.

### Pathway analysis of core proteins and the impact of epigenetic miRNA and DNA methylation on core GEN of stage II HCC

The same analysis procedure as above was also applied to identify the core GEN of stage II HCC, and the results of pathway analysis are summarized in Table 2. The enriched KEGG pathways of stage II HCC included the MAPK signaling, B cell receptor signaling, and toll-like receptor signaling pathways, and cancer-related pathways.

**Table 2:** Pathway analysis of core proteins in the GEN of stage II HCC

Pathway analysis		
**KEGG pathway**	**Numbers**	***p*-value**
**MAPK signaling pathway**	17	2.75E-5
**Pathways in cancer**	16	9.8E-4
**B cell receptor signaling pathway**	7	3.0E-3
**Toll-like receptor signaling pathway**	7	1.1E-2

The enriched KEGG pathways of this GEN included the MAPK signaling pathway, pathways in cancer, B cell receptor signaling pathway, and Toll-like receptor signaling pathway.

**Figure 4:**
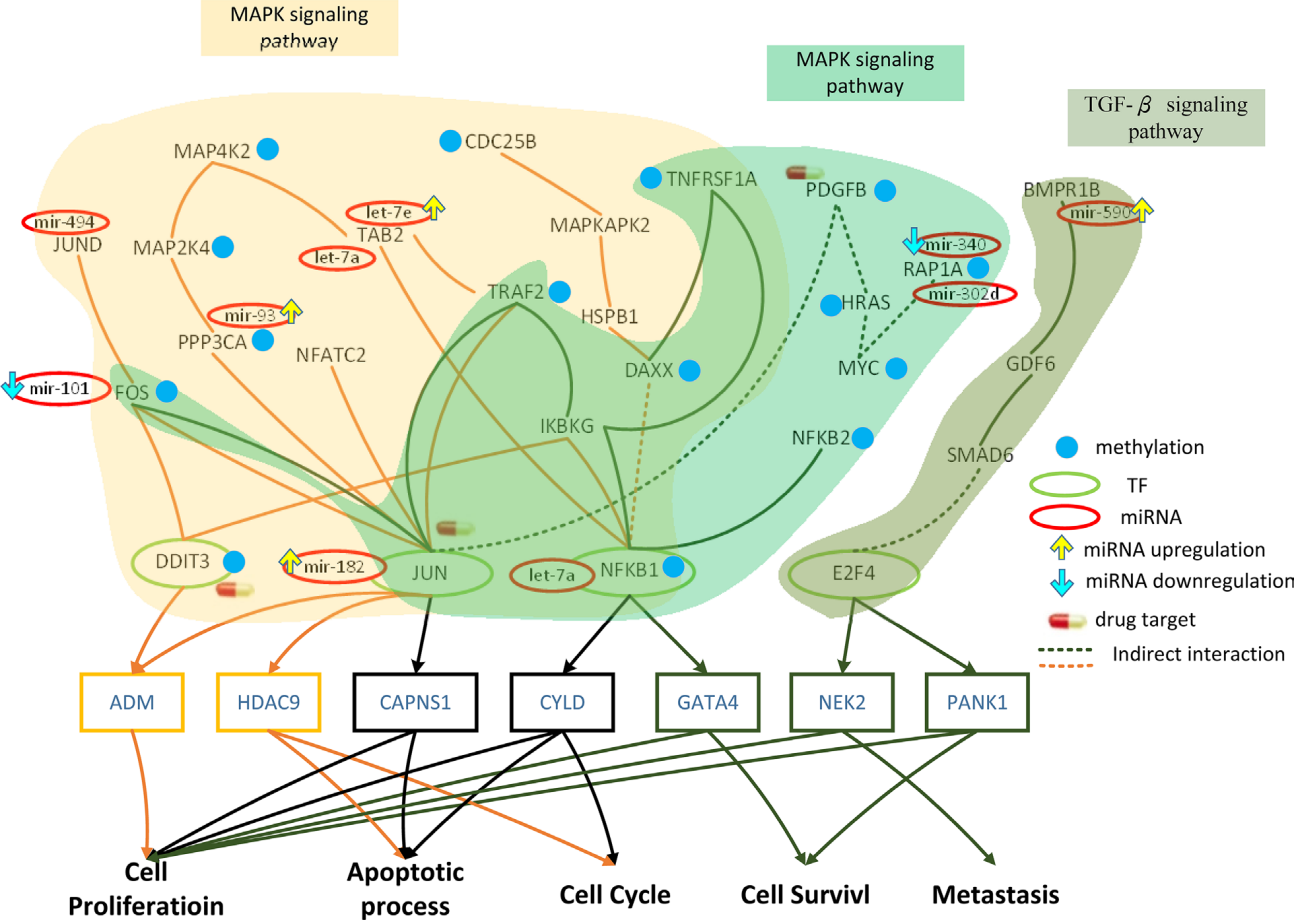
Role of miRNA regulation and DNA methylation on the mechanism of progression of HCC from stage II to stage III The yellow frames and lines represent stage II HCC, while the dark green frames and lines represent stage III HCC. Blue nodes represent DNA methylation. Dark lines and frames represent components observed in both stage II and stage III. The green and red ovals represent transcription factors (TFs) and miRNAs, respectively. The upward yellow arrowhead represents miRNA upregulation during stage III HCC, compared to that in stage II. The downward blue arrowhead represents miRNA downregulation in stage III compared to that in stage II. *TRAF2, MAP2K4, MAP4K2, NFKB1, MAPKAPK2, DAXX, TAB2, DIT3, CDC25B, FOS, JUN, JUND, IKBKG, HSPB1, PPP3CA,* and *NFATC2* are involved in the MAPK signaling pathway during stage II HCC. The transcription factors *DDIT3*, *JUN*, and *NFKB1* are activated by a number of stress signals including the MAPK signaling pathway, which induces cell proliferation, apoptosis, and cell cycle. *TRAF2, TNFRSF1A, FOS, HRAS, PDGFB, JUN, IKBKG, RAP1A, NFKB1, NFKB2,* and *DAXX* are involved in the MAPK signaling pathway, whereas *E2F4, GDF6, SMAD6, PPP2CB,* and *BMPR1B* regulate the TGF-β pathway during stage III HCC. The transcription factors *JUN*, *NFKB1*, and *E2F4* are activated by stress signals, including the MAPK signaling and TGF-β pathways, inducing cellular responses such as cell proliferation, apoptosis, cell cycle, cell survival, and metastasis. miRNA and DNA methylation dysregulation may contribute to the perturbation of the MAPK and TGF-β pathway, resulting in aberrant cellular responses and HCC progression from stage II to stage III. Obviously, the perturbation of the MAPK pathway and the activation of the TGF-β pathway owing to the differences in epigenetic miRNA regulation and DNA methylation may be the major mechanism of HCC progression from stage II to stage III. *DDIT3*, *PDGFB*, and *JUN* were selected as potential drug targets to prevent the progression of HCC from stage II to stage III.

We further identified the TFs DDIT3, JUN, and NFKB1 in the MAPK pathway, encoded by target regions in core genes (Figure 4). This allowed for the analysis of the effect of miRNA and DNA methylation on the MAPK pathway; moreover, we determined that dysfunctions in these processes might contribute to aberrant downstream target gene function.

### Pathway analysis of core proteins and impact of epigenetic miRNA and DNA methylation on core GEN of stage III HCC

The PNP method was used to identify the core GEN of stage III HCC, and the pathways regulated by the core proteins were identified using the DAVID tool. The results of pathway analysis are summarized in Table 3. The enriched KEGG pathways of stage III HCC were the adipocytokine signaling, neurotrophin signaling, MAPK signaling, B cell receptor signaling, and the TGF-β signaling pathways.

**Table 3:** Pathway analysis of core proteins in the GEN of stage III HCC

Pathway analysis		
**KEGG pathway**	**Numbers**	***p*-value**
**MAPK signaling pathway**	12	1.6E-3
**Neurotrophin signaling pathway**	10	7.4E-5
**Adipocytokine signaling pathway**	8	4.9E-5
**B cell receptor signaling pathway**	6	4.7E-3
**TGF-β signaling pathway**	6	8.8E-3

The enriched KEGG pathways included the adipocytokine signaling pathway, neurotrophin signaling pathway, MAPK signaling pathway, B cell receptor signaling pathway, and TGF-β signaling pathway.

We further identified the TFs in various signaling pathways, encoded by target regions in core genes (Figure 4), as follows: *JUN* and *NFKB1* in the MAPK pathway, and *E2F4* in the TGF-β. This allowed us to analyze the effect of miRNA and DNA methylation on the MAPK and TGF-β pathways; we determined that dysfunctions in these processed might cause an aberration in the function of downstream target genes.

### Pathway analysis of core proteins and impact of epigenetic miRNA and DNA methylation on core GEN of stage IV HCC

The PNP method was used to identify the core GEN of stage IV HCC, and the pathways regulated by the core proteins were identified using the DAVID tool. The results of pathway analysis are summarized in Table 4. The enriched KEGG pathways included the adipocytokine signaling, toll-like receptor signaling, Jak-STAT signaling, and the MAPK signaling pathways.

**Table 4:** Pathway analysis of core proteins in the GEN of stage IV HCC

Pathway analysis		
**KEGG pathway**	**Numbers**	***p*-value**
**MAPK signaling pathway**	9	3.0E-3
**Jak-STAT signaling pathway**	7	1.6E-3
**Toll-like receptor signaling pathway**	6	7.4E-5
**Adipocytokine signaling pathway**	5	4.9E-5

The enriched KEGG pathways identified in this GEN are the adipocytokine signaling pathway, Toll-like receptor signaling pathway, Jak-STAT signaling pathway, and MAPK signaling pathway.

We further identified the TFs NFKB1 and STAT3 in the MAPK and Jak-STAT pathways, encoded by target regions in core genes (Figure 4). This allowed for the analysis of the effect of miRNA and DNA methylation on the MAPK and Jak-STAT pathways; moreover, we determined that dysfunctions in these processes might contribute to aberrant downstream target gene function.

### Comparison between the results with the Ingenuity Pathway Analysis and GeneGO Meta-Core software

The Ingenuity Pathway Analysis (IPA 7.0) has been used to identify the core genes, including TF, *APOA1*, *FLNA*, and *HNF4A*, which are associated with HCC by comparing the mRNA expressions of normal liver cells and HCC [21]. *APOA1* has also been identified in the real GENs at four stages of HCC (Figures S1–S4) and in the core GEN of stage III HCC (Figure 2C).

Moreover, the core genes, including *DAPK1*, *BCL2*, *TP53*, and *CCND2*, have also been identified using GeneGO Meta-Core software (Encinitas, CA) to compare the mRNA expressions of normal liver cells and HCC [22]. *DAPK1*, BCL2-like 12 (*BLC2L12*) and p53- inducible gene 3 (*TP53I3*) have also been identified in the real GENs at four stages of HCC (Figures S1–S4). *DAPK1* has been identified in the core GEN of stage I HCC (Figure 2A).

**Figure 5:**
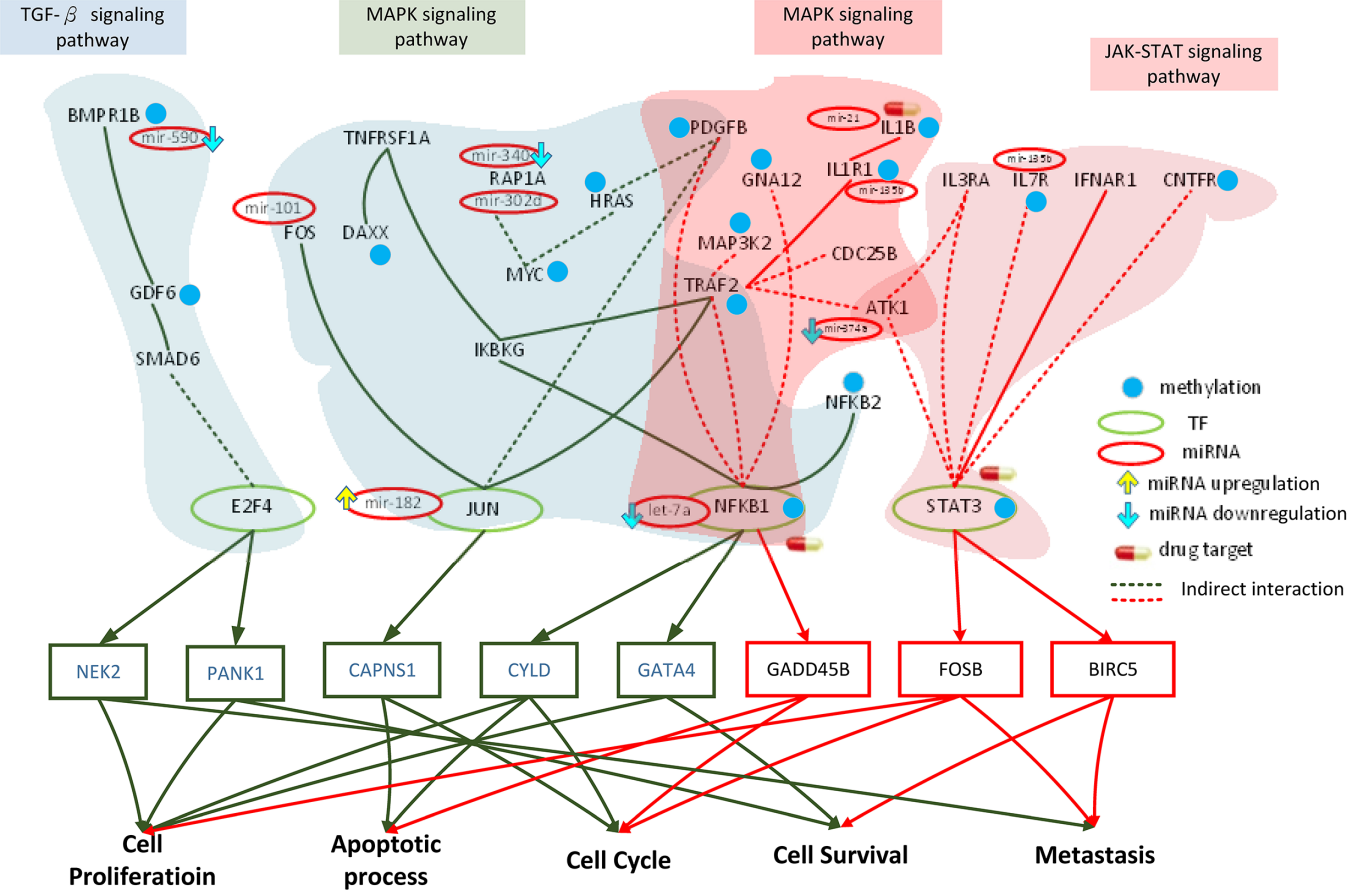
Role of miRNA regulation and DNA methylation on the mechanism of progression of HCC from stage III to stage IV The dark green frames and lines represent stage III HCC. The purple frames and lines represent stage IV HCC. Blue node represents DNA methylation of gene. The green and red ovals represent transcription factors (TFs) and miRNAs, respectively. The upward yellow arrowhead represents miRNA upregulation during stage IV HCC, compared to that in stage III. The downward blue arrowhead represents miRNA downregulation in stage IV compared to that in stage III. *TRAF2, TNFRSF1A, FOS, HRAS, PDGFB, JUN, IKBKG, RAP1A, NFKB1, NFKB2,* and *DAXX* are involved in the MAPK signaling pathway, while *E2F4, GDF6, SMAD6, PPP2CB,* and *BMPR1B* regulate the TGF-β pathway during stage III HCC. The transcription factors *JUN*, *NFKB1*, and *E2F4* are activated by stress signals, including the MAPK signaling and TGF-β pathways, inducing cellular responses such as cell proliferation, apoptosis, cell cycle, cell survival, and metastasis. *AKT1, TRAF2, IL1R1, PDGFB, MAP3K2, GNA12, IL1B, NFKB1,* and *CDC25B* are involved in the MAPK signaling pathway, while *AKT1, CNTFR, IL7R, IL3RA, STAT3,* and *IFNAR1* regulate the JAK-STAT signaling pathway during stage IV HCC. The transcription factors NFKB1 and STAT3 are activated by a number of stress signals, including the MAPK signaling and JAK-STAT pathways, inducing cellular responses, including cell proliferation, apoptosis, cell cycle, cell survival, and metastasis. miRNA and DNA methylation dysregulation may contribute to the perturbation of the MAPK and TGF-β pathways, resulting in aberrant cellular responses and HCC progression from stage III to stage IV. Obviously, the perturbation of the MAPK pathway and the shift from the TGF-β to JAK-STAT pathways caused by epigenetic miRNA regulation and DNA methylation may be the major mechanism of HCC progression from stage III to stage IV. *STAT3*, *ILB*, and *NFKB1* were selected as potential drug targets to prevent the progression of HCC from stage III to stage IV.

Because we applied PNP to the real GENs at four stages of HCC, which contain the identified connection weights using genome-side expression data, to extract the core genes based on the principal structure of GENs, the identified core genes. The purpose of the identification of the core GENs in Figure 2A–2D is to integrate the transcriptionally and epigenetically impaired mechanisms of HCC progression from stage I to II, from stage II to III, and from stage III to IV from a perspective of signal transduction pathways. Thus, we compared the networks between Figure 2A–2B, Figure 2B–2C, and Figure 2C–2D, and extract the genes with the most different connections and top *D*(*k*) involved in significant network structure in (15) from receptors to TFs and their connected miRNAs and genes as the core proteins/genes/miRNAs to consist the core pathways in Figures 3–5. The core pathways from receptors to TFs (Figures 3–5) were obviously different from the core genes identified by high degree of interactions (hub genes) using IPA or GeneGO Meta-Core software.

## DISCUSSION

### Role of epigenetic miRNA regulation and DNA methylation on HCC progression from stage I to stage II

The core GEN in stage I HCC (Figure 2A) were mapped to KEGG pathways, and pathways containing TFs targeting core genes as shown in Figure 3. *AKT1, PTK2, HRAS, CAMK2G, CAMK2D, PI3KR2, CRK*, and *MYC* were found to be involved in the ErbB signaling pathway. Receptors of ErbB trigger a rich network of signaling pathways, culminating in responses ranging from cell division to death and motility to adhesion, which are often dysregulated in cancer [23]. Additionally, *AKT1, TNFRSF1A, HRAS, MAP3K5, MAPT, IKBKG, NTRK2, PPP3R1, RAP1A, PPP3CA, CRK,* and *MYC* were found to be involved in the MAPK signaling pathway. The MAPK signaling pathway regulates a number of cellular activities, including proliferation, differentiation, survival, and death. Dysregulation of the MAPK signaling pathway has been implicated in the development of many human diseases, including various types of cancers [24].

*AKT1*, *HRAS*, and *MYC* are inhibited by the miRNA mir-374a, let-7a, and mir-374a, respectively, in the ErbB signaling pathway (Figure 3). These genes are also inhibited by the same miRNA in the MAPK pathway; additionally, *PPP3R1* and *PPP3CA* are inhibited by mir-148a and mir-93, respectively. Therefore, the dysregulation of these miRNA may interrupt these pathways, inducing aberrant target gene (*MYC*) function.

A comparison of the methylation level between stage I and stage II HCC (Figure 3) revealed that 5 and 2 genes were differentially methylated in the ErbB (*ATK1, PTK2, CAMK2D, CRK,* and *MYC*) and MAPK pathways (*TNFRSF1A* and *NTRK2*), respectively. *AKT1* is involved in cell proliferation and survival [25], while CAMK2D, a member of the CAMK family, activates cancer cell proliferarion [26]. In fact, CRK proteins have been reported to be dysregulated in several human malignancies [27]. MYC, a proto-oncogene, is frequently dysregulated, and is therefore responsible for tumorigenesis in many types of cancer [28].

Extracellular and intracellular stimuli pass through the MAPK and ErbB signaling pathways to MYC, and regulates the cellular process via negative transcriptional regulation of its target genes, including *DUSP1*, *EIF4E*, *PPIA*, and *XPO1* (Figure 3). *DUSP1* is involved in apoptosis, while EIF4E regulates the cell proliferation and cell cycle. PPIA is involved in cell proliferation, apoptosis, and the cell cycle.

The core GEN of stage II HCC (Figure 2B) were mapped to the KEGG pathways, and pathways containing core TFs as well as the target genes were selected (Figure 3). *TRAF2, MAP2K4, MAP4K2, NFKB1, MAPKAPK2, DAXX, TAB2, DIT3, CDC25B, FOS, JUN, JUND, IKBKG, HSPB1, PPP3CA*, and *NFATC2* were found to be involved in the MAPK signaling pathway. The MAPK pathway has been described previously.

Moreover, *NFKB1*, JUND, TAB, and PPP3CA were inhibited by the miRNA let-7a, mir-494, let-7a and let-7e, and mir-93, respectively. Dysregulation of these miRNA may induce changes in these pathways, resulting in aberrant function of the target genes *DDIT3*, *JUN*, and *NFKB1*.

We also found that 6 genes in stage II HCC were differentially methylated compared to that seen in stage I HCC (*TRAF2, MAP2K4, NFATC2, MAPKAPK2, CDC25B,* and *PPP3CA*; Figure 3). TRAF2 is a member of the TRAF protein family, which regulates cell death and cellular response to stress [29]. *MAP2K4*, and *MAPKAPK2* belong to the MAPK family, which is involved in cell proliferation, differentiation, and apoptosis [30], while CDC25B plays an important role in cell cycle, and is deregulated in several types of cancer [31].

Interestingly, although the MAPK signaling pathway also appears in stage II HCC, different TFs are involved in the cellular process. Extracellular and intracellular stimuli pass through the MAPK signaling pathway to *DDIT3*, *JUN*, and *NFKB1*, and thereby regulate the cellular process via negative transcriptional regulation of their target genes, *ADM, HDAC9, CAPNS1,* and *CYLD* (Figure 3). ADM, a reported tumor growth factor, regulates the apoptotic process [32]. HDAC9, a member of HDAC family, is linked to tumor development and regulates cell cycle and apoptosis [33]. CAPNS1 has been reported to play a role in tumor growth and metastasis, and regulates the apoptotic process and proliferation [34]. CYLD, a tumor suppressor, regulates the apoptotic process, proliferation, and cell cycle.

In conclusion, we identified several genes (*AKT1, PTK2, CAMK2D, CRK* and *MYC* in the ErbB pathway and *TNFRSF1A*, *NTRK2*, and *MYC* in the MAPK pathway) and miRNAs (mir-374a, let-7a, mir-148a, and mir-93) that were differentially methylated or dysregulated, causing the perturbation of the MAPK and ErbB pathways, resulting in the aberrant regulation of *MYC* on *DUSP1*, *EIF4E*, *PPIA,* and *XPO1*, and subsequent dysfunction in cellular processes, including cell proliferation, apoptosis, and cell cycle (Figure 3). In addition, the genes *TRAF2, MAP2K4, NFATC2, MAPKAPK2,* and *CDC25B* were differentially methylated and the miRNA let-7a, mir-494, let-7e, and mir-93 were dysregulated during stage II HCC, inducing MAPK pathway perturbation, which in turn resulted in the aberrant regulation of *DDIT3*, *JUN*, *NFKB1* on their corresponding target genes, *ADM*, *HDAC9, CAPNS1,* and *CYLD*, and subsequent dysfunction in cell proliferation, apoptosis, and cell cycle. Obviously, the shift from the ErbB pathway to MAPK pathway (due to different epigenetic miRNA regulation and methylation), triggering changes in the cell responses via different TFs, may be the main mechanism of progression from stage I to stage II HCC.

Multiple drug molecules to prevent the progression of HCC from stage I to stage II based on the target genes of dysregulated miRNA and genes with methylation changes, as shown in Figure 3; moreover, the selected drug molecules should induce minimal side effects on other genes highlighted in Figure 3. The strategy to identify multiple drug molecules to prevent the progression of HCC from stage I to stage II is that we used the Drug-Gene Interaction database (DGIdb) to search drugs, which specifically interact one gene in Figure 3. According to the DGIdb [35], lestaurtinib, dinaciclib, and perifosine specifically interact with *NTRK2*, *MYC,* and *AKT1*, respectively, and have a minimal side effect on the other genes. Additionally, the proposed drug molecules have proven anti-cancer effects [36–38]. Therefore, we designed a drug, comprising lestaurtinib, dinaciclib, and perifosine, to prevent the progression of HCC from stage I to stage II (Table 5).

**Table 5:** Multi-drug design for the prevention of HCC progression from stage I to stage II, based on the progression mechanism elucidated in Figure 6

Drug molecule	Target gene
lestaurtinib	*NTK2*
dinaciclib	*MYC*
perifosine	*AKT1*
Multiple drug molecules	
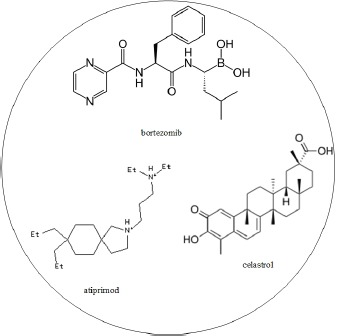	

Lestaurtinib, dinaciclib, and perifosine were proposed to prevent HCC progression from stage I to stage II. The structures of these drug molecules (obtained from the ZINC database) are illustrated in the accompanying figure.

The US Food and Drug Administration (FDA) approved orphan drug status for lestaurtinib and dinaciclib in the treatment of acute myeloid leukemia (AML) and chronic lymphoid leukemia (CLL), respectively. The FDA has approved Fast Track designation for perifosine in the treatment of refractory advanced colorectal cancer.

### Role of epigenetic miRNA regulation and DNA methylation on HCC progression from stage II to stage III

The core genes in the GEN of stage II HCC (Figure 2B) were mapped to the KEGG pathways, and signaling pathways containing core TFs as well as core target genes were selected (Figure 4). *TRAF2, MAP2K4, MAP4K2, NFKB1, MAPKAPK2, DAXX, TAB2, DIT3, CDC25B, FOS, JUN, JUND, IKBKG, HSPB1, PPP3CA*, and *NFATC2* have been found to be involved in the MAPK signaling pathway. The MAPK pathway and the impact of miRNA and DNA methylation on stage II HCC have been detailed in the previous section. In this section, we will focus the effect of miRNA regulation and DNA methylation on the signaling pathway, their corresponding TFs, and target genes of these TFs in stage III HCC.

By comparing the methylation levels between stage II and stage III (Figure 4), we identified 6 genes that were differentially methylated in stage III (*TRAF2, MAP2K4, DAXX, DDIT3, CDC25B,* and *PPP3CA*). DAXX is a multifunctional protein that regulates a wide range of cellular signaling pathways for cell survival and apoptosis [39], while DDIT3 plays a central role in endoplasmic reticulum stress and DNA damage response, by inducing cell cycle arrest and apoptosis [40].

The core GEN of stage III HCC (Figure 2C) was mapped to KEGG pathways, and pathways containing core TFs along with their target genes were selected (Figure 4). *TRAF2, TNFRSF1A, FOS, HRAS, PDGFB, JUN, IKBKG, RAP1A, NFKB1, NFKB2,* and *DAXX* were found to be involved in the MAPK signaling pathway. The MAPK signaling pathway has been detailed in the previous section. Moreover, *E2F4, GDF6, SMAD6, PPP2CB,* and *BMPR1B* were found to be involved in the TGF-β signaling pathway. The transforming growth factor β (TGF-β) superfamily signaling pathways are ubiquitous and essential regulators of cellular processes, including proliferation, differentiation, migration, and survival, as well as physiological pathways [41].

In the MAPK pathway, *FOS*, *JUN*, *PAP1A*, and *NFKB1* are inhibited by mir-101, mir-182, mir-340, let-7a, and *BMPR1B* in the TGF-β pathway is inhibited by the miRNA mir-590. Therefore, deregulations of these miRNA may cause changes in these pathways, resulting in aberrant function of the target genes of *JUN, NFKB1*, and *E2F4*.

In addition, 8 genes were differentially methylated between stage II and III of HCC (*TRAF2, TNFRSF1A, HRAS, PDGFB, NFKB1, DAXX* and *MYC* in the MAPK pathway, and *MYC* in TGF-β pathway). T*RAF2, DAXX,* and *TNFRSF1A* have been described previously. NFKB1 is being increasingly considered as an important TF in cancer progression [42]. HRAS is an essential component of signaling networks controlling cellular proliferation, differentiation, or survival [43].

Extracellular and intracellular stimuli can pass through the MAPK signaling pathway to *JUN* and *NFKB1*, thereby influencing the cellular process through negative transcriptional regulation of their target genes, *CAPNS1*, *CYLD*, and *GATA4* (Figure 4). The functions of *CAPNS1* and *CYLD* have been mentioned in the previous section. *GATA4*, a GATA factor coordinating cellular maturation with proliferation arrest and cell survival, uniquely appears in stage III HCC and is involved in proliferation and cell survival [44]. Moreover, extracellular and intracellular stimuli pass through the TGF-β signaling pathway to *E2F4*, thereby influencing the cellular process through transcriptional regulation of its target genes *NEK2* and *PANK1*. Uncontrolled *NEK2* activity has been reported to activate several oncogenic pathways, thereby inducing cancer cell proliferation and metastasis [45]. *PANK1* regulates cell survival and proliferation.

In conclusion, the methylation of *MAP2K4,* and *DDIT3* and the deregulation of let-7a, mir-494, let-7e, and mir-93 may induce changes in the MAPK pathway in stage II, resulting in aberrant regulation of the target genes of *DDIT3, JUN,* and *NFKB1* (*ADM, HDAC9, CAPNS1, CYLD*) and subsequent dysfunction of cell proliferation, apoptosis, cell cycle, and cell survival (Figure 4). Moreover, the changes in the methylation of *TNFRSF1A, HRAS, PDGFB,* and *MYC* and the deregulation of mir-101, mir-182, mir-340, mir-302d, let-7a, mir-421, and mir-590 during stage III HCC may induce changes in the MAPK and TGF-β pathways, resulting in the aberrant regulation of the target genes of TFs *NFKB1* in the MAPK pathway and*E2F4* in the TGF-β pathway (*CAPNS1, CYLD, GATA4, NEK2, PANK1*), and subsequent dysfunction in cell proliferation, apoptosis, cell cycle, cell survival, and metastasis (Figure 4). The differences in epigenetic miRNA regulation and methylation causes a the shift from the MAPK to the TGF-β pathway, leading to the dysregulation of the target genes of TFs, resulting in favorable conditions for the progression of HCC from stage II to stage III (such as cell proliferation, anti-apoptosis, aberrant cell cycle, cell survival, and metastasis).

Multiple-drug molecules were designed to prevent the progression of HCC from stage II to stage III, based on the target genes of deregulated miRNA and genes with methylation changes (Figure 4); moreover, as described in the previous section, the selected drugs should have a minimal side effect on the other genes highlighted in Figure 4. DGIdb mining identified celecoxib, axitinib, and vinblastine, which interact with *DDIT3*, *PDGFB*, and *JUN*, respectively, with minimal interactions with other genes and proven anti-cancer effects [46–48]. Therefore, we designed a drug molecule comprising celecoxib, axitinib, and vinblastine to prevent the progression of HCC from stage II to stage III (Table 6).

**Table 6:** The multiple drug design for preventing the progression of HCC from stage II and stage III based on the progression mechanism in Figure 7

Drug molecule	Target gene
celecoxib	DDIT3
axitinib	PDGFB
vinblastine	JUN
Multiple drug molecules	
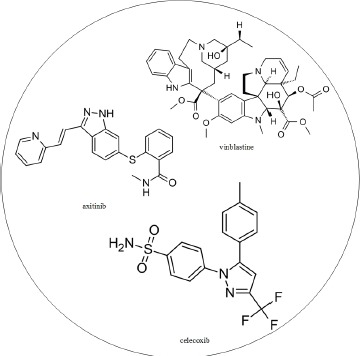	

The multiple drug molecules comprise celecoxib, axitinib, and vinblastine for preventing HCC from stage II to stage III. The structure of these drug molecules based on ZINC database is shown in the following figure.

The US FDA approved celecoxib and axitinib for the relief of the signs and symptoms of Juvenile Rheumatoid Arthritis (JRA) and for the treatment of advanced renal cell carcinoma, respectively. Vinblastine is FDA approved for the treatment of advanced Hodgkin's disease, lymphocytic lymphoma, histiocytic lymphoma, advanced Mycosis fungoides, advanced testicular cancer, Kaposi's sarcoma, and choriocarcinoma.

### Role of epigenetic miRNA regulation and DNA methylation on HCC progression from stage III to stage IV

In stage III of HCC, we map core GEN in Figure 2C to the KEGG pathways and select the pathways containing core TFs simultaneously having core target genes as shown in Figure 5. We found TRAF2, TNFRSF1A, FOS, HRAS, PDGFB, JUN, IKBKG, RAP1A, NFKB1, NFKB2, and DAXX are involved in the MAPK signaling pathway. In addition, E2F4, GDF6, SMAD6, PPP2CB, and BMPR1B are involved in TGF-β pathway. Details of the MAPK pathway and the TGF-β signaling pathway have been described in the previous section. The impact of miRNAs and some DNA methylations on stage III of HCC has also been detailed in the previous section. In this section, we focus on how miRNA regulation and DNA methylation influence the signaling pathways, their corresponding TFs, and target genes of TFs in stage IV of HCC.

By comparing the methylation level between stage III and stage IV of HCC as shown in Figure 5, we found that 8 genes expressed in stage III have differential methylation, i.e. *TRAF2*, *HRAS*, *PDGFB*, *NFKB2*, *DAXX*, *MYC*, *GDF6*, and *BMPR1B*.

In stage IV of HCC, we map core GEN as shown in Figure 2D to the KEGG pathways and select the pathways containing core TFs simultaneously having core target genes as shown in Figure 5. We found AKT1, TRAF2, IL1R1, PDGFB, MAP3K2, GNA12, IL1B, NFKB1, and CDC25B are involved in the MAPK signaling pathway, which is detailed in the above section. We also found AKT1, CNTFR, IL7R, IL3RA, STAT3, and IFNAR1 involved in JAK-STAT signaling pathway. The JAK-STAT signaling pathway is involved in response to cell proliferation, differentiation, cell migration, and apoptosis [49].

In the MAPK pathway of HCC at stage IV, AKT1, IL1R1, IL1B, and NFKB1 are inhibited by mir-374a, mir-135b, mir-21, and let-7a, respectively. In the JAK-STAT pathway, AKT1 is also inhibited by mir-374a and IL7R is inhibited by mir-135b. We consider that the deregulation of these miRNAs may lead to the perturbation of these pathways, resulting in the aberrant function of the target genes coding NFKB1 and STAT3.

By comparing the methylation level between stage III and stage IV of HCC, we found that in stage IV, 8 genes have differential methylation, i.e. *TRAF2*, *IL1R1*, *MAP3K2*, *GNA12*, *IL1B*, *CNTFR*, *IL7R*, and *STAT3* as shown in Figure 5. PDGFB can stimulate growth, survival, and motility of mesenchymal cells [50], which may confer migration ability on tumors. IL7R, a receptor of interleukin 7, can influence the malignancy proliferation by the tumor microenvironment [51]. STAT3 can increase tumor cell proliferation, survival, and invasion, while suppressing anti-tumor immunity [52]. Moreover, IL1R1, MAP3K2, GNA12, IL1B, CNTFR, IL7R, and STAT3 are specific to stage IV of HCC.

Extracellular and intracellular stimuli can pass through the MAPK signaling pathway to NFKB1, and have an impact on the cellular process through the transcriptional regulation of its target gene, including G*ADD45D*. GADD45 is involved in the cell cycle and apoptotic process. Furthermore, extracellular and intracellular stimuli can pass through Jak-STAT signaling pathway to STAT3, and further influence its target genes, including FOSB and BIRC5. Although BIRC5 is mainly involved in cell survival, reportedly, it may provide a beneficial source to fuel tumor cell-invasion and metastasis [53]. FOSB can form a transcription factor complex, AP-1, which can control invasion of tumors [54] and has been reported to promote metastasis in other cancers [55]. Therefore, the deregulation of miRNAs and methylation of target genes would influence the perturbation of these pathways and the subsequent cellular dysfunctions consequently promoting the progression of HCC from stage III to stage IV, as shown in Figure 5.

In conclusion, at stage III of HCC, we found that the methylation of 6 genes (i.e. *HRAS*, *NFKB2*, *DAXX*, *MYC*, *GDF6*, and *BMPR1B*) and downregulation of 7 miRNAs (i.e. mir-101, mir-182, mir-340, mir-302d, let-7a, mir-421, and mir-590) to their target genes may contribute to perturbation of the MAPK pathway and the TGF-β pathway, resulting in the aberrant regulation of TFs (i.e. NFKB1 in MAPK pathway and E2F4 in TGF-β pathway) on their corresponding target genes (i.e. *CAPNS1*, *CYLD*, *GATA4*, *NEK2*, and *PANK1*) and subsequent dysfunctions in cell proliferation, apoptosis, cell cycle, cell survival, and metastasis, as shown in Figure 5. In addition, in stage IV, we found that the methylation of 7 genes (i.e. *IL1R1*,*MAP3K2*, *GNA12*, *IL1B*, *CNTFR*, *IL7R*, and *STAT3*) and deregulation of 5 miRNAs (i.e. mir-374a, mir135b, mir-21, let-7a, and mir-135a) to their target genes may contribute to perturbation of the MAPK pathway and the JAK-STAT pathway, resulting in the aberrant regulation of TFs (i.e. NFKB1 in MAPK pathway and STAT3 in JAK-STAT pathway) on their corresponding target genes (i.e. *GADD45B*, *FOSB*, and *BIRC5*) and the subsequent dysfunctions in cell proliferation, apoptosis, cell cycle, cell survival, and metastasis as shown in Figure 5. Owing to different epigenetic miRNA regulation and DNA methylation, there is a shift from the TGF-β pathway to the JAK-STAT pathway and different proteins involved in the MAPK pathway cause the downsregulation of TFs to their respective target genes, thus regulating cellular responses (i.e. cell proliferation, apoptotic process, cell cycle, cell survival, and metastasis) and subsequently causing the progression of HCC from stage III to stage IV, as shown in Figure 5.

Multiple-drug molecules were designed based on the target genes of dysregulating miRNAs and genes exhibiting differential methylation, to prevent the progression of HCC from stage III to stage IV, as shown in Figure 5. The selection of drugs should have a minimal side effect on other genes. According to DGIdb, atiprimod, celastrol, and bortezomib interact with STAT3, IL1B, and NFKB1, respectively, and have minimal interaction with other genes. Additionally, the proposed multiple-drug molecules have exhibited anti-cancer effects [56–58]. Therefore, we have designed a multiple-drug molecule comprising atiprimod, celastrol, and bortezomib, to prevent the progression of HCC from stage III to stage IV. The multiple-drug design is shown in Table 7.

**Table 7:** The multiple drug design for preventing the progression of HCC from stage III and stage IV based on the progression mechanism in Figure 8

Drug molecule	Target gene
atiprimod	STAT3
celastrol	IL1B
bortezomib	NFKB1
Multiple drug molecules	
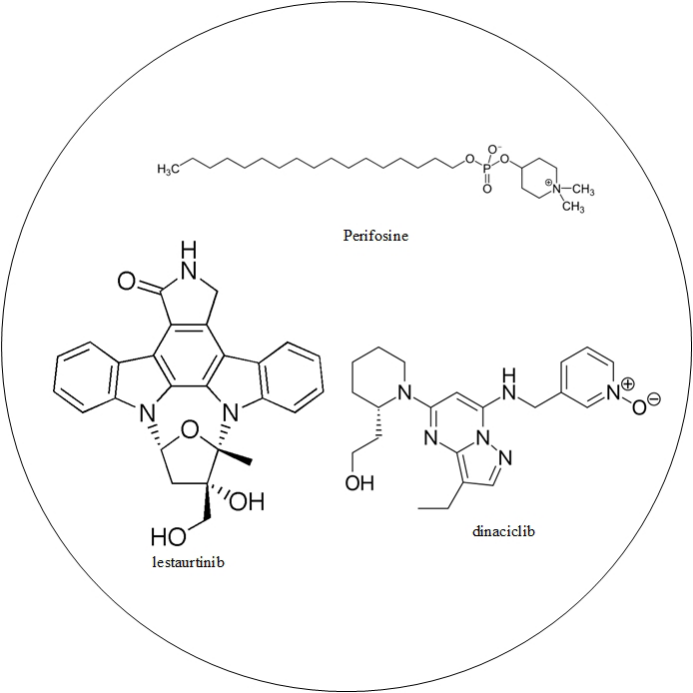	

The multiple drug molecules comprise atiprimod, celastrol, and bortezomib for preventing HCC from stage III to stage IV. The structure of these drug molecules based on ZINC database is shown in the following figure.

Atiprimod was granted orphan drug designation by FDA for the treatment of multiple myeloma. FDA approved bortezomib for initial treatment of patients with multiple myeloma.

### Overall mechanism of HCC progression and identified HCC-specific drugs

**Figure 6:**
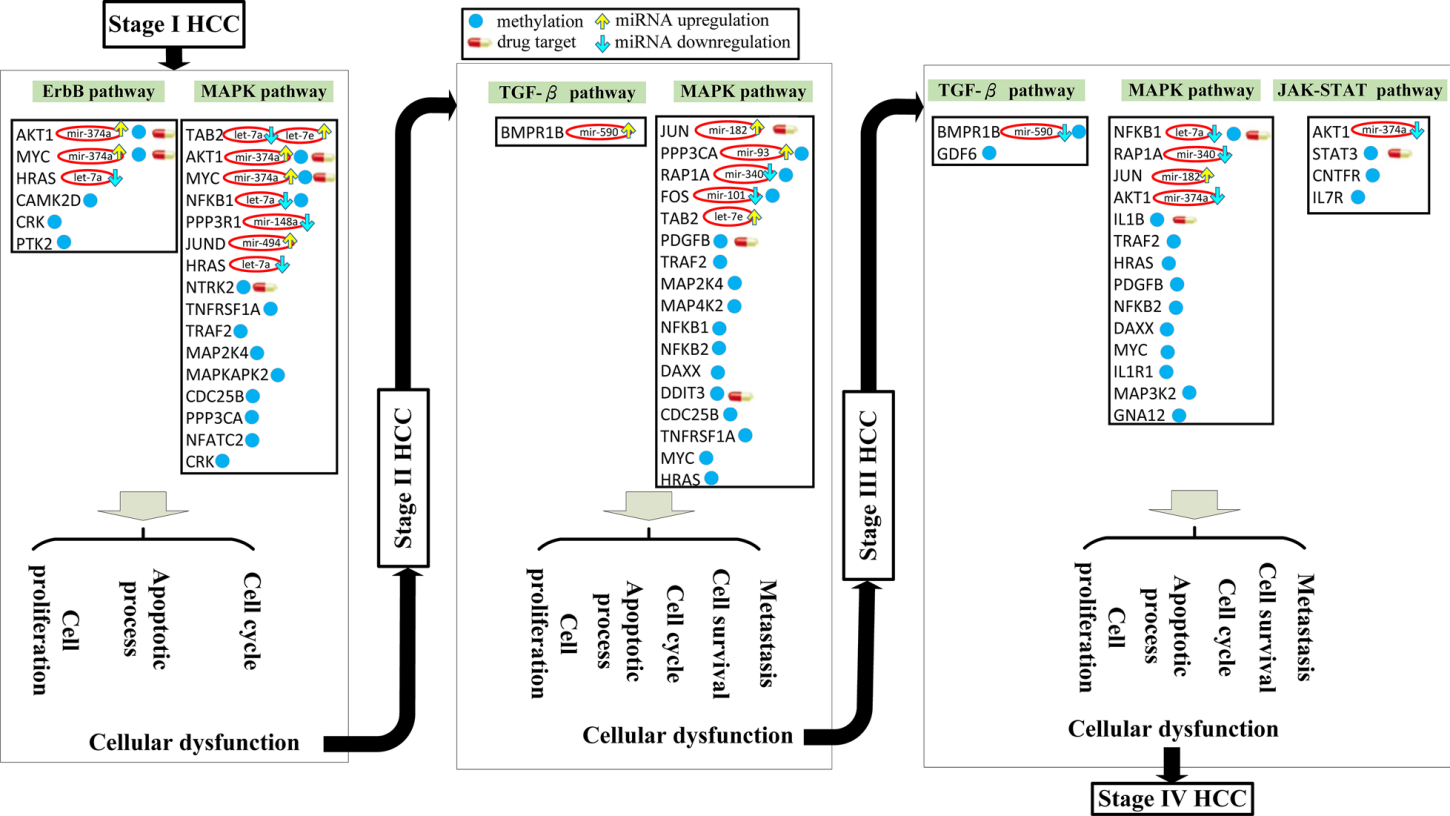
Overall mechanism of HCC progression from stage I to stage IV via miRNA deregulation and DNA methylation Figures 3 to 5 summarize the overall mechanism of HCC progression from stage I to stage IV. The pathway members are all influenced by miRNA regulation and/or epigenetic DNA methylation; the members in brown were only affected by miRNA. miRNA deregulation and DNA methylation lead to distortion of the ErbB and MAPK signaling pathways, inducing distortions in the cell cycle, apoptosis, and cell proliferation processes, resulting in the progression of HCC from stage I to stage II. Similarly, induced distortions in the MAPK and TGF-β signaling pathway caused metastasis and cell cycle, apoptosis, and cell proliferation dysfunction, resulting in HCC progression from stage II to stage III. miRNA dysregulation and DNA methylation changes were also responsible for distortions in the MAPK, TGF-β, and JAK-STAT signaling pathways, resulting in metastasis and cell cycle, apoptosis, and cell proliferation dysfunction, which in turn induces HCC progression from stage III to stage IV. As a whole, miRNA and DNA methylation dysregulation of the various downstream genes in tumor cells could induce changes in the various signaling pathways, resulting in aberrant cell response, which favors HCC progression.

The overall progression (summarized from Figure 3 to Figure 5) is shown in Figure 6. Signaling pathways are perturbed by the dysregulation of miRNA and methylation changes in the transduction process. Signal transduction of extracellular and intracellular stimuli is distorted by the perturbed signaling pathways, which result in HCC progression. Most of miRNA-regulated or methylated genes and miRNAs including *AKT1* [59, 60], *BMPR1B* [61], *FOS* [62], *GDF6* [63], *HRAS* [59, 62], *IL1R1* [63], *IL7R* [61], *JUND* [60], *MAP2K4* [61], *MYC* [62, 63], *NFATC2* [59], *NFKB1* [63], *NTRK2* [63], *PDGFB* [59], *PPP3CA* [63], *PTK2* [62], *STAT3* [64], let-7e [65], mir-148a [65], mir-494 [66], mir-182 [65], mir-590 [67], mir-340 [65], and mir-93 [65] in Figure 6 can be supported by large-scale analysis studies of DNA methylation data or gene/miRNA expression data in HCC.

According to the results, we identified the MAPK signaling pathway involved in the core GENs of the 4 stages of HCC (Figures 3–5). It has been reported that the MAPK pathway could play an important role in HCC tumor progression and apoptosis [68, 69]. We found 6 specific proteins (NTRK2, MAPKAPK2, JUND, CRK, PPP3R1, and NFATC2) of the MAPK pathway, which participate in the progression of HCC from stage I to stage II. It has been shown that NTRK2 [70], MAPKAPK2 [71], and JUND [72] were associated with the impaired proliferation and CRK [73] was involved in cell adhesion and migration during early HCC. PPP3R1 has been also identified as a diagnostic marker, which mediates the dysregulations of 10 different pathways (natural killer cell mediated cytotoxicity, B cell receptor signaling pathway, T cell receptor signaling pathway, long-term potentiation, calcium signaling pathway, apoptosis, axon guidance, Wnt signaling pathway, and vascular endothelial growth factor (VEGF) signaling pathway), to affect liver immune system, and liver cell proliferation, adhesion and migration in liver carcinogenesis [74]. The dysregulated NFATC2 was involved in the immune cell infiltration to HCC [65]. The nature and localization of immune cells infiltrating the tumor cells have an important impact on tumor progression [75]. Therefore, the differentially methylated specific genes (*NTRK2*, *MAPKAPK2*, *CRK*, and *NFATC2*) and the dysregulated miRNAs (mir-148a, mir-374a and mir-494) in the MAPK and ErbB pathways cause cell proliferation, adhesions and migration and the immune cell infiltration to cancer cells during early HCC.

We found 3 specific proteins (MAP4K2, FOS, and DDIT3) of the MAPK pathway, which participate in the progression of HCC from stage II to stage III. It has been proposed that the proteins MAP4K2 and FOS [76] and the protein DDIT3 [77] mediated the transcriptionally and epigenetically dysregulated proliferation of HCC, respectively. Therefore, the differentially methylated specific genes (*MAP4K2*, *FOS*, and *DDIT3*) and the dysregulated miRNAs (mir-101, and mir-590) in the MAPK and TGF-β pathways cause the transcriptionally and epigenetically impaired signal transduction leading to liver carcinogenesis.

We found 3 specific proteins (GNA12, IL1R1, and MAP3K2) of the MAPK pathway, which participate in the progression of HCC from stage III to stage IV. It has been shown that GNA12 [78], and IL1R1 [79] mediated metastasis of HCC through cytoskeletal changes and autocrine and paracrine mechanisms, respectively. MAP3K2 mediated the methylation-induced tumorigenesis of HCC [80]. Therefore, the differentially methylated specific genes (*GNA12*, *IL1R1*, and *MAP3K2*) and the dysregulated miRNAs (mir-135b, mir-374a, and mir-590) in the MAPK, JAK-STAT, and TGF-β pathways cause the methylation-induced liver tumor growth, HCC cell migration and metastatic spread.

In summary, The differentially expressed miRNAs, mir-148a, mir-374a, mir-497, let-7a and let-7e, and the differentially methylated genes, *AKT1*, *CAMK2D*, *CDC25B*, *CRK*, *HRAS*, *JUND*, *MAP2K4*, *MAPKAPK2*, *MYC*, *NFATC2*, *NFKB1*, *NTRK2*, *PPP3CA*, *PPP3R1*, *TAB2*, *TNFRSF1A* and *TRAF2*, regulate cell proliferation, apoptotic process and cell cycle through ErbB and MAPK pathways in the switch between stage I HCC and stage II HCC.

The differentially expressed miRNAs, mir-93, mir-101, mir-182, mir-340, mir-590 and let-7e, and the differentially methylated genes, *BMPR1B*, *CDC25B*, *DAXX*, *DDIT3*, *FOS*, *JUN*, *MAP2K4*, *MAP4K2*, *MYC*, *NFKB1*, *NFKB2*, *PDGFB*, *PPP3CA*, *RAP1A*, *TAB2*, *TNFRSF1A* and *TRAF2*, regulate cell proliferation, apoptotic process, cell cycle, cell survival and metastasis through TGF-beta and MAPK pathways in the switch between stage II HCC and stage III HCC.

The differentially expressed miRNAs, mir-182, mir-340, mir-374a, mir-590 and let-7a, and the differentially methylated genes, *AKT1*, *BMPR1B*, *CNTFR*, *DAXX*, *GDF6*, *GNA12*, *HRAS*, *IL1B*, *IL1R1*, *IL7R*, *JUN*, *MAP3K2*, *MYC*, *NFKB1*, *NFKB2*, *PDGFB*, *RAP1A*, *STAT3* and *TRAF2*, regulate cell proliferation, apoptotic process, cell cycle, cell survival and metastasis through TGF-beta, MAPK and JAK-STAT pathways in the switch between stage III HCC and stage IV HCC. We have also proposed potential multi-drug molecules for targeted therapy of HCC: multi-drug molecules comprising lestaurtinib, dinaciclib, and perifosine; axitinib, vinblastine, and celecoxib; and atiprimod, celastrol, and bortezomib were designed to target *NTRK2, MYC,* and *AKT1*; *DDIT3, PDGFB,*and *JUN*; and *DDIT3, PDGFB,* and *JUN*, to prevent HCC progression from stage I to II, II to III, and III to IV, respectively.

## MATERIALS AND METHODS

### Overview of the construction of core GENs involved in HCC

The construction of core GENs regulating the 4 stages of HCC is summarized in Figure 1. The NGS profiles of HCC and normal tissue samples, and the miRNA and DNA methylation data were mined from the miRNA, TF-gene, and BioGRID databases to construct candidate GENs composed of interactive candidate GRN, PPIN, and miRNA-gene regulatory networks. The false positive candidate GENs were pruned to construct real GENs affecting the 4 stages of HCC by system modeling and least square estimation method; furthermore, the cancer profiles were classified according to the stages to obtain the GENs for each stage, using the corresponding NGS data. The core GENs for each stage were obtained by PNP; the PNP method was used to extract the significant structure of GENs to investigate the mechanism of progression of HCC. The core GENs were mapped to pathways in the DAVID database and compared to those of the neighboring stages to investigate the mechanism of progression of hepatocarcinogenesis. We also propose potential drugs against genes responsible for significant epigenetic changes to prevent hepatocarcinogenesis using the Drug Gene Interaction database (DGIdb).

### Data selection and processing

The original data from the Cancer Genome Atlas (TCGA) was downloaded to, and processed in, the user-friendly UCSC Cancer Genomics browser [81]. In this study, the level-3 gene expression, miRNA expression, and DNA methylation profiles were used to construct GRNs and PPINs. One American Indian, 148 Asian, 15 African American, 156 Caucasian, and 18 patients of undefined ethnicity were included in this study. The disease stage was classified according to the TNM (T: size of tumors, ranging from T1 to T4; N: nearby lymph node metastasis, with N0 and N1 representing no lymph node metastasis and lymph node metastasis, respectively; M: distant metastasis, with M0 and M1 representing distant and no metastasis, respectively) staging system into stages I, II, IIIA, IIIB, IIIC, IVA, and IVB. Patients classified into stages IIIA, IIIB, and IIIC were combined into a single stage III group, and those in stage IVA and IVB were combined into a group named stage IV. The patients were divided into stages based on the available clinical data. In addition to the 50 samples from normal subjects, the dataset comprised 163, 83, 80, and 6 tissue samples from subjects with HCC stage I, II, III, and IV, respectively.

Candidate GENs were enriched using information from the starBase [82] and miRTarBase [83] (for miRNA-gene regulatory associations), Gene Set Enrichment Analysis (GSEA) [84], Human Transcription Regulation Interaction database (HTRIdb) [85] and integrated transcription factor platform (ITFP) [86] (for TF-gene regulatory associations), and Biological General Repository for Interaction Datasets (BioGRID) [87] (for protein-protein interactions) databases, in order to construct candidate GENs.

Database mining leads to the enrichment of candidate GENs with a large number of false positives; these were pruned in the candidate GENs by deleting insignificant components in the system order (the number of interactions at each node in the GEN).

### Modeling GENs

The regulation of each gene combines transcriptional and epigenetic regulation, and includes the negative regulation of miRNA and the effect of promoter methylation on the target gene *i* in the candidate GRN; the gene expression of the *i-*th gene in the candidate GRN is described as follows:

$$\begin{array}{lll} x_{i,n} & = & \sum_{\begin{array}{c} \begin{array}{c} j=1\\ j\neq i \end{array} \end{array}}^{J_{i}}a_{\text{ij}}y_{j,n}h(\beta_{i,n})-\sum_{m=1}^{M_{i}}b_{\text{im}}s_{m,n}x_{i,n}+k_{i}\cdot h(\beta_{i,n})\\ & & +v_{i,n},h(\beta_{i,n})=\frac{1}{1+{\left(\beta_{i,n}/0.5\right)}^{2}} \end{array}$$ (1)

where the subscript *n* represents the *n*-th sample; *x_i,n_* represents the expression level of the *i-*th target gene; *y_j,n_* represents the expression level of the *j*-th TF binding to target gene *i*; *s_m,n_* denotes the expression of the *m-*th miRNA; *k_i_* represents the basal level of the target gene *i*; β_*i,n*_ represents the methylation level of the *i*-th target gene (range: 0 to 1, with the value close to zero indicating a low level of methylation, and a value closer to 1 indicating a high level of methylation); *h(β_i,n_)* represents the effect of methylation level β_*i,n*_ on the gene expression of target gene *i*, wherein a higher value decreases the binding affinity *a_ij_* of *J_i_* transcription factors with the target gene *i*; and *v_i,n_* represents the stochastic noise caused by other factors and model uncertainty. The biological meaning of equation (1) is described as follows: the mRNA expression of target gene *i* is regulated by *J_i_* transcription factors binding to the target gene *i,* wherein methylation modification *h(β_i,n_)* decreases the binding effectiveness of TF (*a_ij_*); mRNA expression is also negatively regulated by the partial or perfect binding *b_im_* of miRNA *s_m,n_* to the mRNA of target gene *i*. Moreover, the basal level *k_i_* of mRNA expression could also be affected by the methylation modification *h(β_i,n_)*. Some stochastic noise was employed to model the measurement noise and random fluctuations of expression in target gene *i*.

The level of expression of the protein *p* in the candidate GEN was described using the following equation in the candidate PPIN:

$$y_{p,n}=\sum_{\begin{array}{l} q=1\\ q\neq i \end{array}}^{Q_{p}}c_{\text{pq}}y_{q,n}y_{p,n}+e_{p}x_{p,n}+h_{p}+w_{p,n}$$ (2)

where the subscript *n* represents the *n-*th sample; *y_p,n_* represents the protein expression of protein *p*; *y_q,n_* represents the *q-*th protein interacting with protein *p*; *e_p_* denotes the translation rate of the corresponding mRNA of the *p-*th protein; *h_p_* represents the basal level of protein *p*; *c_pq_* represents the interaction ability between *q-*th and *q-*th protein; and *w_p,n_* represents the stochastic noise caused by other factors or model uncertainty. The interaction ability of PPI is proportional to the product of concentrations of both proteins, that I, it is proportional to the probability of molecular collisions between them. Biologically, equation (2) indicates that the protein expression level of protein *p* is determined by complex protein interactions with *Q_p_* proteins, in addition to the basal level of protein *p* and some stochastic noise.

### Construction of GEN using the system identification method and system order detection

The candidate GEN obtained by big database mining includes all possible associations under various environmental conditions; therefore, their actual associations must be further confirmed by applying the gene expression, miRNA expression, and methylation profiles in the NGS data to the identification of the following regression models. The false-positive candidate GENs were pruned by deleting the insignificant components from the system order through system identification and system order detection in (1) and (2).

The equation (1) can be rewritten as the following regression form

$$\begin{array}{lll} x_{i,n} & = & [y_{l,n}h(\beta_{i,n})\cdots y_{J_{i},n}h(\beta_{i,n})-s_{l,n}x_{i,n}\\ & & \cdots -s_{{\text{M}}_{i},n}x_{i,n}h(\beta_{i,n})]\left[\begin{array}{c} a_{i1}\\ \vdots \\ a_{iJ_{i}}\\ b_{i1}\\ \vdots \\ b_{iM_{i}}\\ k_{i} \end{array}\right]+v_{i,n}\\ & = & \phi_{i,n}\theta_{i}^{GRN}+v_{i,n} \end{array}$$ (3)

where *ϕ*_*i,n*_ is the regression vector which can be obtained from the corresponding data in NGS and $\theta_{i}^{\text{GRN}}$ is the parameter vector to be estimated for target gene *i*.

The equation (3) with *L* patients can be arranged as the following form

$$\left[\begin{array}{c} x_{i,1}\\ x_{i,2}\\ \vdots \\ x_{i,L} \end{array}\right]=\left[\begin{array}{c} \phi_{i,1}\\ \phi_{i,2}\\ \vdots \\ \phi_{i,L} \end{array}\right]\theta_{i}^{\text{GRN}}+\left[\begin{array}{c} v_{i,1}\\ v_{i,2}\\ \vdots \\ v_{i,L} \end{array}\right]$$ (4)

where *L* is the number of corresponding patients. For convenience, we define the notation *Χ_i_*, *Φ_i_*, and *V_i_* to represent (4)

$$X_{i}=\Phi_{i}\theta_{i}^{\text{G}RN}+V_{i}$$ (5)

The constrained least square estimation of $\theta_{i}^{\text{GRN}}$ is formulated as

$$\underset{{}_{\theta_{i}^{\text{GRN}}}}{\min}\|X_{i}-\Phi_{i}\theta_{i}^{\text{GRN}}{\|}_{2}\text{ subject to }\forall mb_{im}\geq 0\;and\;k_{i}\geq 0$$ (6)

Similarly, equation (2) can be rewritten as the following form

$$\begin{array}{lll} y_{p,n} & = & \left[y_{1,n}y_{p,n}\;\cdots \;y_{Q_{p},n}y_{p,n}\;x_{p,n}\;1\right]\left[\begin{array}{c} c_{p1}\\ \vdots \\ c_{pQ_{p}}\\ e_{p}\\ h_{p} \end{array}\right]+w_{p,n}\\ & = & \psi_{p,n}\theta_{p}^{PPI}+w_{p,n} \end{array}$$ (7)

where *ψ_p,n_* denotes regression vector with the corresponding data and $\theta_{p}^{PPI}$ is parameter vector to be estimated. The equation (7) with *L* patients can be arranged as the following form

$$\left[\begin{array}{c} y_{p,1}\\ y_{p,2}\\ \vdots \\ y_{p,L} \end{array}\right]=\left[\begin{array}{c} \psi_{p,1}\\ \psi_{p,2}\\ \vdots \\ \psi_{p,L} \end{array}\right]\theta_{p}^{PPI}+\left[\begin{array}{c} w_{p,1}\\ w_{p,2}\\ \vdots \\ w_{p,L} \end{array}\right]$$ (8)

and equation (8) also can be simplified by defining *Υ_p_*, *Ψ_p_*, and *W_p_* as the following equation

$$Y_{p}=\Psi_{p}\theta_{p}^{PPI}+W_{p}$$ (9)

The constrained least square parameter identification problem is formulated below

$$\underset{{}_{\theta_{p}^{PPI}}}{\min}\|Y_{p}-\Psi_{p}\theta_{p}^{PPI}{\|}_{2}\text{ subject to }h_{p}\geq 0$$ (10)

We then applied the function *lsqlin* in MATLAB optimization toolbox to the constrained least square parameter identification problems of GRN in (6) and PPI in (10) to solve the binding affinity of TF *j* to gene *i*, *a_ij_*, the repression ability of miRNA *m* to gene *i*, −*b_im_*, and the interaction ability between protein *p* and protein *q*, *c_pq_*, subject to −*b_im_* ≤ 0, *k_i_* ≥ 0, and *h_p_* ≥ 0 to meet the practical situation. Akaike information criterion (AIC) is accompanied with the parameter identification procedures for determining system model orders (i.e. *J*_i_, *M*_i_ and *Q_p_*) in (1) and (2) for each protein in candidate PPIN, each gene in candidate GRN and each miRNA in candidate miRNA regulatory network in candidate GENs at 4 stages of HCC [88]. Then we prune the false-positive gene and miRNA regulations (i.e. *a_ij_* and *b_im_*) and protein interactions (i.e. *c_pq_*) out of true system model orders determined by AIC, and hence the real gene transcription regulations, miRNA regulations and protein interactions are obtained through real NGS data for real GENs at different stages of HCC as shown in Figure S1 to Figure S4.

### Remark

According to the formulated problem in (3), (6), (7), and (10), if we want to identify the general models of GRN in (1) and PPIN in (2) with the consideration of translation process from mRNA to protein, the simultaneously measured expressions of the genome-wide mRNAs and proteins at four stages of HCC were required. Since genome-wide expression measurement of protein behaviors at four stages of HCC have not been realized yet and mRNA expressions are proportional to their corresponding proteins, in which 73% variance of protein abundance can be explained by mRNA abundance [89], the mRNA expressions can replace protein expressions for the above constrained least square parameter estimation problems in (6), and (10). In the parameter estimation problem of PPIN in (2), if we replace protein expression *y_p,n_* by its corresponding mRNA expression *x_p,n_*, it is impossible to identify the translation rate *e_p_* (if time-course microarray data is used, this kind of problem can be avoided.). In this situation, the parameter estimation of PPIN in (10) is with the modified $\psi_{p,n}=\left[y_{1,n}y_{p,n}\;\cdots \;y_{Q_{p},n}y_{p,n}\;1\right]$ and the modified $\theta_{p}^{PPI}=\left[\begin{array}{c} c_{p1}\\ \vdots \\ c_{pQ_{p}}\\ h_{p} \end{array}\right]$ in (9) and (10). It means that if we did not have the simultaneously measured expressions of the genome-wide mRNAs and proteins at four stages of HCC, the translation rate of the corresponding mRNA to the *p-*th protein, *e_p_*, can not be identified by solving the problem in (10). In future, if the genome-wide expressions of mRNAs and proteins are available, we could construct a more complete integrated network, including translation paths in hepatocarcinogenesis.

### Extracting core GENs of different stages of HCC using Principal Network Projection

The actual GENs of each stage are very complex; therefore, it is not easy to gain insights into the most significant mechanisms of tumor development and progression from these GENs during different stages of HCC. Therefore, the core GENs at the different stages of HCC were extracted using PNP based on PCA. Although SVD has been a popular tool for a dimensionality reduction method by performing a PCA on the given network matrix, it can not guarantee parameter polarity, i.e. the miRNA repressions −*b_im_* ≤ 0, in the network matrix. Therefore, we only applied PCA to the identified network matrix to determine the core nodes in the network based on the projection distance, which only deleted the insignificant nodes and did not change the connection weights between core nodes. The dimensionality reduction of the network matrix is called PNP method.

### Let the combined network matrix of GEN

$$H={\left[\begin{array}{ccccccccccccccc} c_{1,1} & \cdots & c_{1,j} & \cdots & c_{1,N} & a_{1,1} & \cdots & a_{1,j} & \cdots & a_{1,N} & -b_{1,1} & \cdots & -b_{1,j} & \cdots & -b_{1,M}\\ \vdots & \ddots & \vdots & \ddots & \vdots & \vdots & \ddots & \vdots & \ddots & \vdots & \vdots & \ddots & \vdots & \ddots & \vdots \\ c_{j,1} & \cdots & c_{j,j} & \cdots & c_{j,j} & a_{j,1} & \cdots & a_{j,j} & \cdots & a_{j,N} & -b_{j,1} & \cdots & -b_{j,j} & \cdots & -b_{j,M}\\ \vdots & \ddots & \vdots & \ddots & \vdots & \vdots & \ddots & \vdots & \ddots & \vdots & \vdots & \ddots & \vdots & \ddots & \vdots \\ c_{N,1} & \cdots & c_{N,j} & \cdots & c_{N,N} & a_{N,1} & \cdots & a_{N,j} & \cdots & a_{N,N} & -b_{N,1} & \cdots & -b_{N,j} & \cdots & -b_{N,M} \end{array}\right]}^{T}$$ (11)

where *c_i,j_* is the interaction ability between protein *i* and *j* protein in (2), *a_i,j_* is the regulatory ability of TF *j* on gene *i* in (1), and −*b_i,j_* is the epigenetic regulation of miRNA *j* on gene *i* in (1). The combined network matrix of GEN can be decomposed by singular value decomposition (SVD) as follows [88, 90–94]

$$H=U\times D\times V^{T}$$ (12)

where $U\in {ℜ}^{(2N+M)\times N},\;V\in {ℜ}^{N\times N},h_{k,:}$ denotes the *k-th* row vector of H for k=1,⋯,(2N+M), and *v_:,m_* denote the *m-th* column vector of *V,* for *m* = 1, ⋯, *N*, which are right singular vectors of *H.* The diagonal entries $d_{i},\;i=1,2,\cdots ,N$ of the diagonal matrix, $D=diag(d_{1},\cdots ,d_{m},\cdots ,d_{N})$, are the N singular values of *H* in descent order, i.e. $d_{1}\geq d_{2}\geq \cdots \geq d_{N}$. The eigenexpression fraction (*E_m_*) is defined as

$$E_{m}=\frac{d_{m}^{2}}{\sum_{n=1}^{N}d_{n}^{2}}$$ (13)

We selected the top *M* singular vectors of *V* such that $\sum_{m=1}^{M}d_{m}^{2}\geq 0.85$ with the minimal *M,* so that the *M* principal components contain 85% of the combined network matrix from an energy point of view. The projections of *H* onto the *M* singular vectors of *V* are defined as follows

$$\begin{array}{lll} P(k,m) & = & h_{k,:}\times v_{:,m}\;for\;k=1,\\ & & \cdots ,(2N+M)\text{ and }m=1,\cdots ,M \end{array}$$ (14)

We further defined the 2-norm distance from each gene to the top *M* singular vectors.

$$\begin{array}{lll} D(k) & = & {\left[\sum_{m=1}^{M}\left[P(k,m)\right]\right]}^{1/2},\\ & & for\;k=1,\cdots ,(2N+M) \end{array}$$ (14)

If *D(k)* is close to zero, it implies that the *k-th* gene is independent of the top *M* singular vectors. Hence, we define three thresholds, *th*_1_, *th*_2_, *th*_3_ to respectively identify the core proteins *D(k)* ≥ *th*_1_ for k=1,…,*N,* and the core genes *D*(*k*) ≥ *th*_2_ for k = *N* + *1*,…,2*N*, and the core miRNAs *D*(*k*) ≥ *th_3_* for k = 2*N*,…,2*N + L,* in the core GEN of the integrated genetic and epigenetic network, which has the principal network structure of the GEN.

## CONCLUSIONS

In this study, we constructed GENs specific for the various stages (I–IV) of HCC, based on their corresponding mRNA, miRNA, and methylation profiles in NGS data, in order to elucidate the mechanism of disease progression, through big database mining via least square estimation and AIC model order detection. Core GRNs were obtained to identify core network markers by PNP method, based on PCA. The involvement of the pathways utilized by the PPIs of core GENs at each stage in the mechanism of progression was identified using DAVID. The core network markers of pathways were compared among the neighboring stages to identify the impact of the changes in DNA methylation and miRNA regulation of the ErbB, MAPK, TGF-β, and JAK-STAT signaling pathways on hepatocarcinogenesis. We discovered that these signals were further transduced to different TFs to aberrantly regulate their target genes, resulting in the development of favorable factors (cell proliferation, anti-apoptosis, cell cycle, cell survival and, especially in stage III and stage IV, metastasis) for the progression and development of HCC. Moreover, we proposed multi-drug molecules targeting multiple drug target genes with aberrant methylation and deregulating miRNA, to prevent the progression of HCC from a systematic perspective. Multi-drug molecules comprising lestaurtinib, dinaciclib, and perifosine; axitinib, vinblastine, and celecoxib; and atiprimod, celastrol, and bortezomib were designed to target *NTRK2, MYC,* and *AKT1*; *DDIT3, PDGFB,* and *JUN*; and *DDIT3, PDGFB,* and *JUN*, to prevent HCC progression from stage I to II, II to III, and III to IV, respectively

## SUPPLEMENTARY MATERIALS


