# Supplementary material for: Investigating the mechanism of hepatocellular carcinoma progression by constructing genetic and epigenetic networks using NGS data identification and big database mining method
Source: Oncotarget. 2016 Nov 4;7(48):79453–73. doi: 10.18632/oncotarget.13100 (PMC5346727; doi:10.18632/oncotarget.13100)
Supplement: Supplementary file 1 [file oncotarget-07-79453-s001.pdf]

Investigating the mechanism of hepatocellular carcinoma progression by constructing genetic and epigenetic networks using NGS data identification and big database mining method

Supplementary Materials

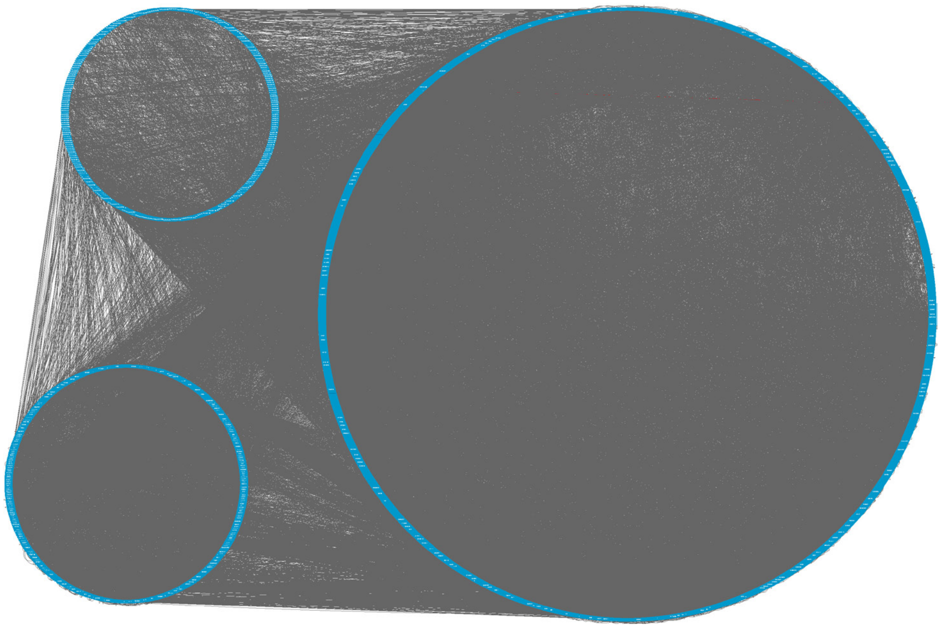

Supplementary Figure S1: The genetic and epigenetic network (GEN) of HCC at stage I. The upper left circle represents the miRNA (326), the lower left circle represents the TFs (408) and the big right circle represents the proteins (3741) for the GEN of HCC at stage I. Links within the “proteins” circle represent proteins-proteins interactions, links between the “TFs” circle and the “proteins” circle represent the regulations of transcription factors on target genes and links between the “miRNAs” circle and the “proteins” circle represent the regulations of miRNAs on target genes in the GEN of HCC at stage I.

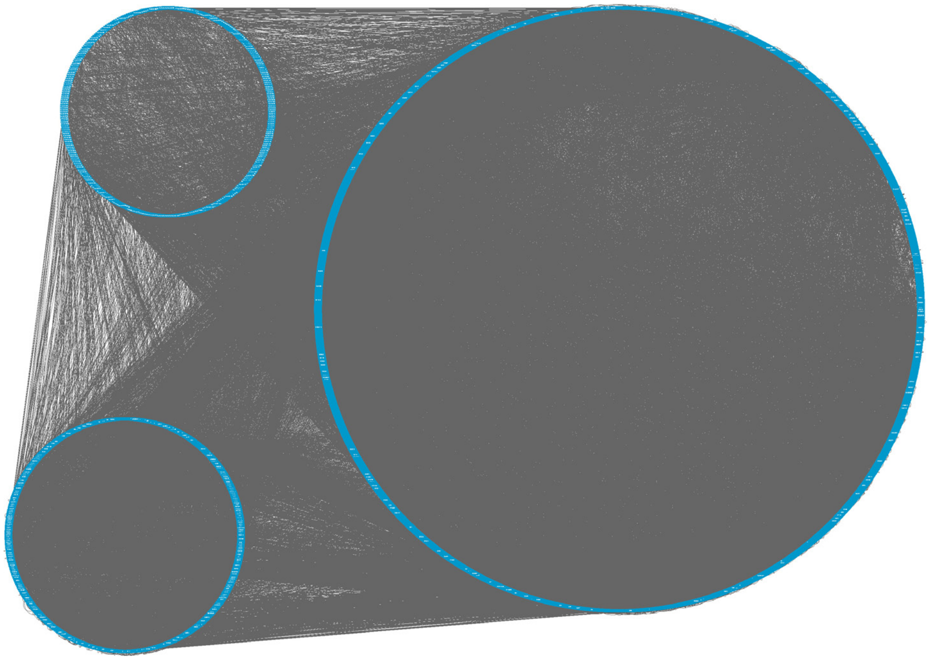

Supplementary Figure S2: The genetic and epigenetic network (GEN) of HCC at stage II. The upper left circle represents the miRNA (326), the lower left circle represents the TFs (408) and the big right circle represents the proteins (3741) for the GEN of HCC at stage II. Links within the “proteins” circle represent proteins-proteins interactions, links between the “TFs” circle and the “proteins” circle represent the regulations of transcription factors on target genes and links between the “miRNAs” circle and the “proteins” circle represent the regulations of miRNAs on target genes in the GEN of HCC at stage II.

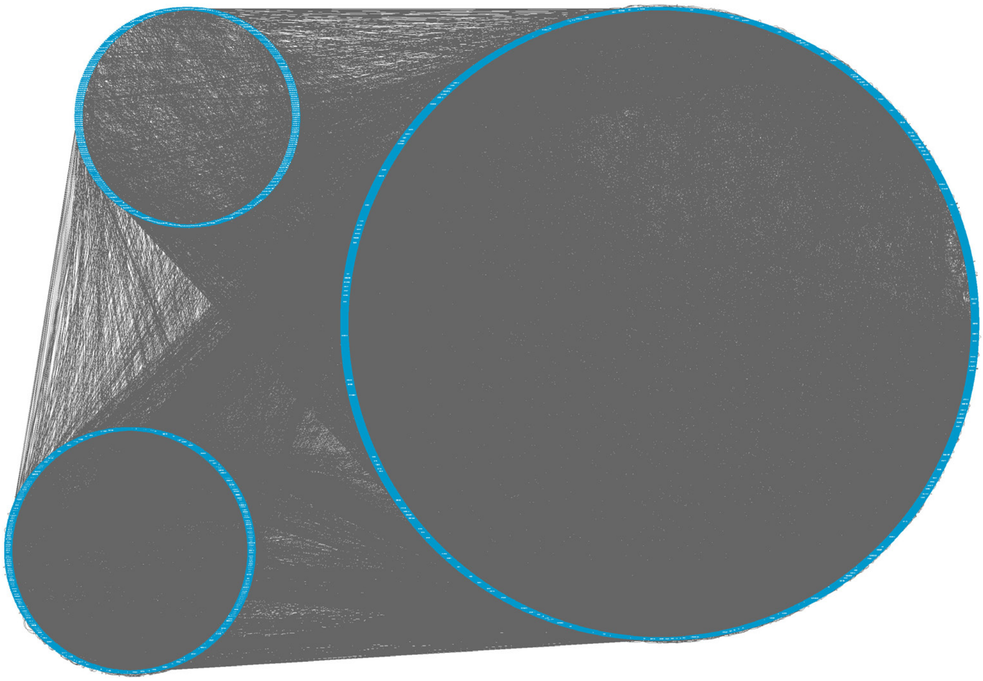

Supplementary Figure S3: The genetic and epigenetic network (GEN) of HCC at stage III. The upper left circle represents the miRNA (326), the lower left circle represents the TFs (408) and the big right circle represents the proteins (3741) for the GEN of HCC at stage III. Links within the “proteins” circle represent proteins-proteins interactions, links between the “TFs” circle and the “proteins” circle represent the regulations of transcription factors on target genes and links between the “miRNAs” circle and the “proteins” circle represent the regulations of miRNAs on target genes in the GEN of HCC at stage III.

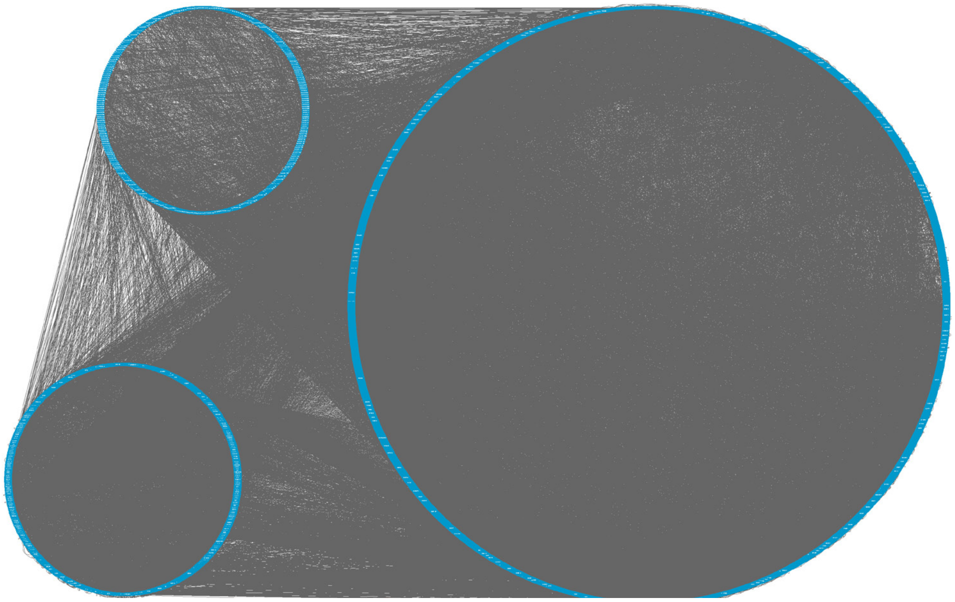

Supplementary Figure S4: The genetic and epigenetic network (GEN) of HCC at stage IV. The upper left circle represents the miRNA (326), the lower left circle represents the TFs (408) and the big right circle represents the proteins (3737) for the GEN of HCC at stage IV. Links within the “proteins” circle represent proteins-proteins interactions, links between the “TFs” circle and the “proteins” circle represent the regulations of transcription factors on target genes and links between the “miRNAs” circle and the “proteins” circle represent the regulations of miRNAs on target genes in the GEN of HCC stage at IV.

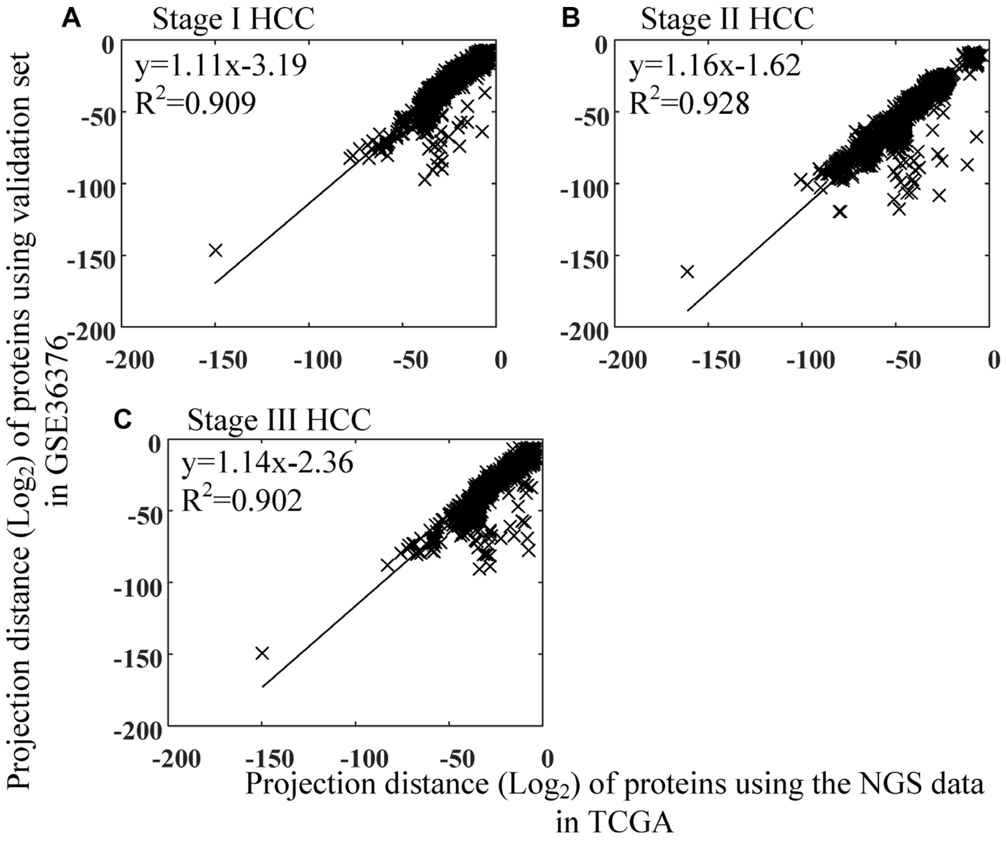

Supplementary Figure S5: The relationship between the projection results identified using the data in GSE36376 and TCGA.

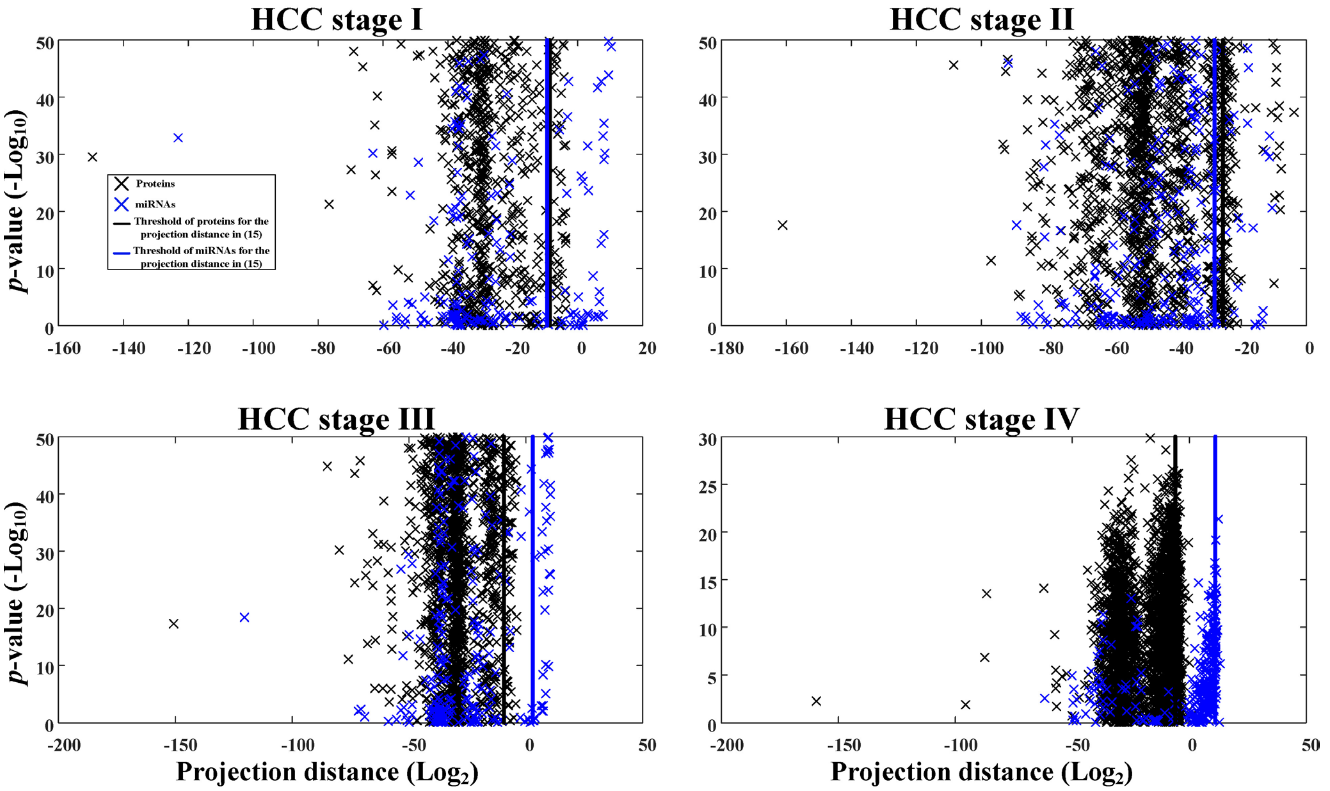

Supplementary Figure S6: The plot of p-value (log10) versus projection distance (log2) of each component in HCC stage 1–4 (Supplementary Figures S1–S4).
